# Preparation of Half- and Post-Metallocene Hafnium Complexes with Tetrahydroquinoline and Tetrahydrophenanthroline Frameworks for Olefin Polymerization

**DOI:** 10.3390/polym11071093

**Published:** 2019-06-27

**Authors:** Jun Won Baek, Su Jin Kwon, Hyun Ju Lee, Tae Jin Kim, Ji Yeon Ryu, Junseong Lee, Eun Ji Shin, Ki Soo Lee, Bun Yeoul Lee

**Affiliations:** 1 Department of Molecular Science and Technology, Ajou University, Suwon 16499, Korea; 2 Department of Chemistry, Chonnam National University, 77 Yongbong-ro, Buk-gu, Gwangju 500-757, Korea; 3 LG Chem, Ltd., 188, Munji-ro, Yuseong-gu Daejeon 305-738, Korea

**Keywords:** post-metallocene, half-metallocene, hafnium complex, olefin polymerization, coordinative chain transfer polymerization

## Abstract

Hafnium complexes have drawn attention for their application as post-metallocene catalysts with unique performance in olefin polymerization. In this work, a series of half-metallocene HfMe_2_ complexes, bearing a tetrahydroquinoline framework, as well as a series of [N^amido^,N,C^aryl^]HfMe_2_-type post-metallocene complexes, bearing a tetrahydrophenanthroline framework, were prepared; the structures of the prepared Hf complexes were unambiguously confirmed by X-ray crystallography. When the prepared complexes were reacted with anhydrous [(C_18_H_37_)_2_N(H)Me]^+^[B(C_6_F_5_)_4_]^−^, desired ion-pair complexes, in which (C_18_H_37_)_2_NMe coordinated to the Hf center, were cleanly afforded. The activated complexes generated from the half-metallocene complexes were inactive for the copolymerization of ethylene/propylene, while those generated from post-metallocene complexes were active. Complex bearing bulky isopropyl substituents (**12**) exhibited the highest activity. However, the activity was approximately half that of the prototype pyridylamido-Hf Dow catalyst. The comonomer incorporation capability was also inferior to that of the pyridylamido-Hf Dow catalyst. However, **12** performed well in the coordinative chain transfer polymerization performed in the presence of (octyl)_2_Zn, converting all the fed (octyl)_2_Zn to (polyolefinyl)_2_Zn with controlled lengths of the polyolefinyl chain.

________________________________________________________________________________________

## 1. Introduction

**Scheme 1:**
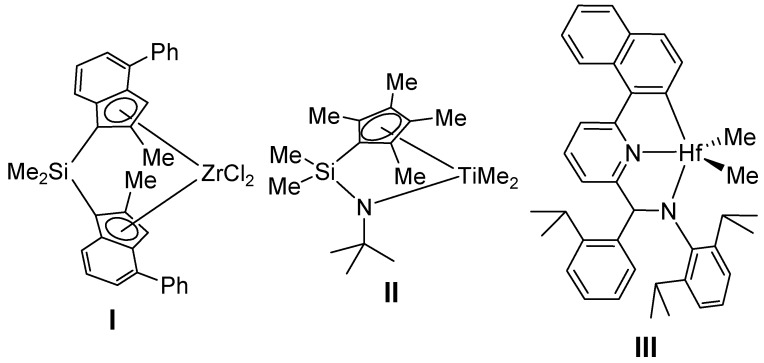
Typical examples of metallocene, half-metallocene, and post-metallocene catalysts.

Polyolefins (POs) are the most abundant polymers, which are mostly produced using the Ziegler-Natta catalyst. The conventional heterogeneous multi-site Ziegler-Natta catalyst has been replaced with homogeneous single-site catalysts, although the former is still a main player in the production of POs. The use of homogeneous single-site catalysts originated with the serendipitous discovery of methylaluminoxane (MAO) by Kaminsky [1]. The initial Zr-based metallocene catalysts, Ti-based half-metallocenes, and post-metallocenes with non-cyclopentadienyl ligands, were developed successively (Scheme 1) [2,3]. A typical example of half-metallocenes is [Me_2_Si(η^5^-Me_4_C_5_)(N*^t^*Bu)]TiCl_2_, which was discovered in the early 1990s at Dow (**II** Scheme 1) [4]. The Ti-based half-metallocenes characteristically exhibit, similar to the Zr-based metallocene catalysts, higher α-olefin incorporation in ethylene/α-olefin copolymerizations, which enables the commercial production of polyolefin elastomers (POE). A typical example of post-metallocenes is the pyridylamido-Hf complex (**III** in Scheme 1), which was discovered in the early 2000s also at Dow [5,6]. The pyridylamido-Hf complex exhibits excellent α-olefin incorporation capability [7], and is capable of controlling the tacticity in the propylene polymerization to produce isotactic-polypropylene [8,9,10]. A unique characteristic of **III** is that the β-elimination process—an intrinsic chain transfer process that inevitably occurs during the olefin polymerization performed with the conventional Zr-based metallocene and Ti-based half metallocene—can be completely prevented [11]. The DFT (density functional theory) calculation results suggest that the β-hydrogen transfer reaction is disfavored by the absence of agostic hydrogen interactions, due to the less acidic nature of the hafnium center [9,10]. In contrast, the agostic hydrogen interaction plays a significant role in the typical Zr-based metallocene catalysis [12]. Absence of the β-elimination process enables the construction of high-molecular-weight polyolefin chains with various block compositions [13]. When polymerization is performed in the presence of a dialkylzinc (e.g., Et_2_Zn), polymer chains are uniformly grown from dialkylzinc due to the rapid alkyl exchange between Zn and the Hf sites; this is called the coordinative chain transfer polymerization (CCTP) [14,15,16]. CCTP is judiciously utilized in the commercial production of olefin block copolymer (OBC) at Dow [5,17,18,19]. It was also demonstrated that the CCTP involving **III**, could be switched to anionic styrene polymerization to prepare polyolefin-polystyrene block copolymers [11,20,21,22]. In this context, many thorough studies have been performed to detail **III**, and to improve the catalytic activity by modifying its ligand skeleton [23,24,25,26,27,28,29,30,31,32]. To develop an upgraded catalyst relative to **III**, we prepared various Hf complexes. Hafnium catalysts typically exhibit low α-olefin incorporation in ethylene/α-olefin copolymerizations and, in this work, ligands were designed to minimize steric hindrance around the reaction site.

## 2. Materials and Methods

All the experiments were performed in an inert atmosphere using a standard glove box and Schlenk techniques. Toluene, hexane, and THF were distilled from benzophenone ketyl. Methylcyclohexane (anhydrous grade) used for the polymerization reactions was purchased from Tokyo Chemical Industry (TCI, Tokyo, Japan) and purified over a Na/K alloy. Sublimed-grade HfCl_4_ was purchased from Strem Chemicals (Newburyport, MA, USA) and used as received. The ethylene/propylene mixed gas was purified over trioctylaluminum (0.6 M in methylcyclohexane), in a bomb reactor (2.0 L). The ^1^H NMR (600 MHz) and ^13^C NMR (150 MHz) analyses were performed on a JEOL ECZ 600 instrument (Tokyo, Japan). Elemental analyses were performed at the Analytical Center of Ajou University (Suwon, South Korea). The GPC data were obtained in 1,2,4-trichlorobenzene, at 160 °C, using a PL-GPC 220 system equipped with a RI detector and two columns (PLgel mixed-B 7.5 × 300 mm from Varian (Polymer Lab, Palo Alto, CA, USA)). The ligand precursors for **1**–**6** [33,34] and compounds **7**–**8** [35] were prepared according to the reported method.

### 2.1. Preparation of **1**

MeMgBr (2.60 mL, 3.11 M in diethyl ether) was added dropwise to a stirred solution of 8-(tetramethylcyclopentadienyl)-1,2,3,4-tetrahydroquinoline (0.500 g, 1.97 mmol) and THF (7 mL) at room temperature. The solution was stirred for 6 h at 60 °C, and the generated methane gas was vented off, simultaneously. After cooling to room temperature, HfCl_4_ (0.632 g, 1.97 mmol) was added to the resulting solution. After the solution was stirred for 12 h at room temperature, the solvent was removed using a vacuum line. The residue was extracted with hexane (4 mL × 6). The removal of the solvent produced a yellow solid, which was pure according to the results of the ^1^H and ^13^C NMR spectra analyses (Figure S1 in Supporting Information; 0.794 g, 87%). An analytical pure compound, containing single crystals that are suitable for X-ray crystallography, was obtained by recrystallization in hexane at −30 °C. ^1^H NMR (C_6_D_6_): δ 7.05 (d, *J* = 7.8 Hz, 1H), 6.96 (d, *J* = 6.6 Hz, 1H), 6.82 (t, *J* = 7.2 Hz, 1H), 3.80 (t, *J* = 4.8 Hz, 2H), 2.50 (t, *J* = 6.6 Hz, 2H), 2.00 (s, 6H), 1.78 (s, 6H), 1.62 (q, *J* = 6 Hz, 2H), −0.02 (s, Hf(CH_3_)_2_ 6H) ppm. ^13^C{^1^H} NMR (C_6_D_6_): δ 10.80, 22.94, 27.19, 43.31, 48.35, 116.67, 118.73, 122.10, 125.57, 129.17, 130.78, 160.69 ppm. Anal. Calcd. (C_20_H_27_HfN): C, 52.23; H, 5.92; N, 3.05%. Found: C, 52.21; H, 5.91; N, 3.08%.

### 2.2. Preparation of **2**

Complex **2** was prepared by the same procedure and experimental conditions as those employed for **1**, using 2-methyl-8-(tetramethylcyclopentadienyl)-1,2,3,4-tetrahydroquinoline (0.420 g, 1.57 mmol). The reaction between the ligand precursor and MeMgBr was so slow that 30 h was required for the reaction to reach completion. A yellow solid compound was obtained (0.594 g, 80%). ^1^H NMR (C_6_D_6_): δ 7.06 (d, *J* = 7.2 Hz, 1H), 7.00 (d, *J* = 7.0 Hz, 1H), 6.82 (t, *J* = 6.6 Hz 1H), 4.15 (m, 1H, NCH), 2.64 (m, 1H), 2.47 (t, *J* = 5.4 Hz, 1H), 2.00 (s, 6H, CH_3_), 1.80 (s, 3H, CH_3_), 1.75 (s, 3H, CH_3_), 1.48–1.56 (m, 2H), 1.32 (d, *J* = 6.0 Hz, 3H, NCCH_3_), −15.2 (s, 3H, HfCH_3_), −27.8 (s, 3H, HfCH_3_) ppm. 13C{1H} NMR (C_6_D_6_): δ 10.66, 10.87, 10.88, 11.01, 21.50, 23.68, 29.08, 47.40, 47.52, 49.83, 116.31, 117.70, 118.55, 121.32, 125.51, 129.28, 130.15, 130.32, 159.54 ppm. Anal. Calcd. (C_21_H_29_HfN): C, 53.22; H, 6.17; N, 2.96%. Found: C, 53.22; H, 6.14; N, 2.98%.

### 2.3. Preparation of **3**

Complex **3** was prepared by the same procedure and experimental conditions as those employed for **1**, using 8-(2,4,5-trimethyl-6H-cyclopenta[b]thiophen-6-yl)-1,2,3,4-tetrahydroquinoline (0.420 g, 1.36 mmol). A light brown solid compound was obtained. An analytical pure compound, containing single crystals that are suitable for X-ray crystallography, was obtained by recrystallization in hexane at −30 °C (0.530 g, 62%). ^1^H NMR (C_6_D_6_): δ 7.15 (dd, *J* = 6.6 Hz, 1H), 6.95 (dd, *J* = 7.8 Hz, 1H), 6.78 (t, *J* = 6.6 Hz, 1H), 6.41 (quartet, *J* = 1.2 Hz, 1H, SCCH), 3.80 (m, 1H), 3.75 (m, 1H), 2.46 (t, *J* = 6.0 Hz, 2H), 2.20 (s, 3H, CH_3_), 2.10 (d, *J* = 1.8 Hz, 3H, SCCH_3_), 1.60 (m, 2H), 0.07 (s, 3H, HfCH_3_), −0.29 (s, 3H, HfCH_3_) ppm. ^13^C{^1^H} NMR (C_6_D_6_): δ 11.30, 11.58, 16.21, 22.73, 27.12, 43.13, 49.72, 50.53, 106.21, 116.51, 117.20, 118.80, 122.42, 124.57, 129.77, 129.94, 134.89, 136.11, 143.96, 160.81 ppm. Anal. Calcd. (C_21_H_25_HfNS): C, 50.25; H, 5.02; N, 2.79; S, 6.39%. Found: C, 50.21; H, 5.01; N, 2.78; S, 6.39%.

### 2.4. Preparation of **4**

Complex **4** was prepared by the same procedure and experimental conditions as those employed for **1**, using 2-methyl-8-(2,4,5-trimethyl-6H-cyclopenta[b]thiophen-6-yl)-1,2,3,4-tetrahydroquinoline (0.420 g, 1.36 mmol). The reaction between the ligand precursor and MeMgBr was so slow that 30 h was required for the reaction to reach completion. A light brown solid compound was obtained. An analytically pure compound was obtained through recrystallization in hexane at −30 °C (0.467 g, 67%). ^1^H NMR (C_6_D_6_): δ 7.17 and 7.15 (d, *J* = 6.3 Hz, 1H), 6.99 and 6.98 (d, *J* = 8.4 Hz, 1H), 6.79 (t, *J* = 7.8 Hz, 1H), 6.42 and 6.38 (d, *J* = 1.2 Hz, 1H, SCCH), 4.21 and 4.14 (m, 1H, NCH), 2.66 and 2.63 (m, 1H), 2.44 and 2.41 (dt, *J* = 4.8 Hz, H), 2.21 and 2.20 (s, 3H, CH_3_), 2.12 and 2.11 (s, 3H, SCCH_3_), 1.85 and 1.79 (s, 3H, CH_3_), 1.55(m, 2H), 1.3 and 1.27 (d, *J* = 7.2 Hz, 3H, SCCH_3_), 0.09 and 0.08 (s, 3H, HfCH_3_), −0.28 and −0.30 (s, 3H, HfCH_3_) ppm. ^13^C{^1^H} NMR (C_6_D_6_): δ 11.12, 11.41, 11.58, 11.76, 16.12, 16.23, 21.20, 21.35, 23.07, 23.54, 28.50, 28.73, 46.63, 47.07, 48.71, 49.69, 51.34, 51.71, 105.88, 107.45, 116.47, 116.59, 116.85, 118.62, 121.41, 121.65, 124.63, 129.12, 129.83, 129.92, 131.20, 134.42, 134.67, 135.29, 135.68, 143.86, 144.41 159.47, 159.61 ppm. Anal. Calcd. (C_22_H_27_HfNS): C, 51.21; H, 5.27; N, 2.71; S, 6.21%. Found: C, 51.20; H, 5.24; N, 2.73; S, 6.22%.

### 2.5. Preparation of **5**

Complex **5** was prepared by the same procedure and experimental conditions as those employed for **1**, using fluorenyltetrahydroquinoline (0.194 g, 0.651 mmol). The product was marginally soluble in hexane and it was extracted with toluene (2 mL × 3). An analytically pure compound was obtained through recrystallization in hexane, at −30 °C (0.161 g, 49%). ^1^H NMR (C_6_D_6_): δ 7.88 (d, *J* = 8.4 Hz, 2H), 7.34 (d, *J* = 6.6 Hz, 1H), 7.22 (d, *J* = 8.4 Hz, 2H), 7.01 (t, *J* = 7.2 Hz, 2H), 6.94 (t, *J* = 8.4 Hz, 2H), 6.91 (t, *J* = 7.2 Hz, 1H), 3.60 (m, 2H, NCH_2_), 2.53 (t, *J* = 6.0 Hz, 2H) 1.56 (m, 2H), −0.69 (s, 6H, Hf(CH_3_)_2_) ppm. ^13^C{^1^H} NMR (C_6_D_6_): δ 22.51, 27.22, 42.89, 52.53, 103.54, 114.32, 118.91, 121.16, 121.84, 122.58, 123.58, 125.13, 128.60, 129.88, 130.24, 136.63, 159.98 ppm. Anal. Calcd. (C_24_H_23_HfN): C, 57.20; H, 4.60; N, 2.78%. Found: C, 57.16; H, 4.59; N, 2.79%.

### 2.6. Preparation of **6**

Complex **6** was prepared by the same procedure and experimental conditions as those employed for **1**, using 2-methyl-8-(fluorenyl)tetrahydroquinoline (0.154 g, 0.494 mmol). The product was marginally soluble in hexane and it was extracted with toluene (2 mL × 3). An analytical pure compound, containing single crystals that are suitable for X-ray crystallography was obtained by recrystallization in hexane at −30 °C (0.158 g, 62%). ^1^H NMR (C_6_D_6_): δ 7.88 (d, *J* = 8.4 Hz, H), 7.86 (d, *J* = 8.4 Hz, H), 7.34 (d, *J* = 7.2 Hz, H), 7.24 (d, *J* = 9.0 Hz, H), 7.18 (d, *J* = 8.4 Hz, H), 7.12 (d, *J* = 7.8 Hz, H), 7.05 (t, *J* = 7.8 Hz, H), 7.01 (t, *J* = 7.2 Hz, H), 6.93 (m, 3H), 4.01 (m, H), 2.73 (m, H), 2.48 (dt, *J* = 4.8 Hz, H), 1.52 (m, 2H), 1.09 (d, *J* = 6.6 Hz, 3H, NCCH_3_), −0.68 (s, 3H, HfCH_3_), −0.71 (s, 3H, HfCH_3_) ppm. ^13^C{^1^H} NMR (C_6_D_6_): δ 20.78, 22.89, 28.05, 45.96, 51.32, 54.09, 103.13, 113.66, 115.63, 118.80, 120.85, 120.90, 121.48, 122.42, 122.94, 123.68, 124.86, 125.54, 128.70, 129.99, 130.30, 135.85, 136.20, 158.46 ppm. Anal. Calcd. (C_25_H_25_HfN): C, 57.97; H, 4.87; N, 2.70%. Found: C, 57.89; H, 4.79; N, 2.73%.

### 2.7. Preparation of **9**

Isopropyllithium (6.13 mL, 0.70 M in pentane) was slowly added to a stirred suspension of 2-phenyl-1,10-phenanthroline (1.00 g, 3.90 mmol) in toluene (10 mL), at −30 °C. The solution was heated to 0 °C and stirred for 35 min. H_2_O (16 mL) was added and the organic compounds were extracted with CH_2_Cl_2_ (3 × 10 mL). The solvent was removed using a vacuum line and the residue redissolved in CH_2_Cl_2_ (10 mL). Activated MnO_2_ (3.68 g, 42.3 mmol) was added, and the solution was stirred for 12 h under atmospheric exposure. After filtration over anhydrous MgSO_4_, the solvent was removed with a rotary evaporator. Pure 2-isopropyl-9-phenyl-1,10-phenanthroline was obtained by column chromatography on silica gel, using ethyl acetate/hexane (1/10, *v*/*v*) (1.01 g, 87%). The prepared 2-isopropyl-9-phenyl-1,10-phenanthroline (1.01 g, 3.39 mmol), Ru(OTf)(TsDPEN)(η^6^-*p*-cymene) (TfO = trifluoromethanesulfonate, TsDPEN = *N*-toluenesulfonyl-1,2-diphenylethylenediamine) (0.17 mmol), and MeOH (17 mL) were added to a bomb reactor. After charging H_2_ to 50 bar, the reaction mixture was stirred for 12 h at room temperature. After releasing H_2_, it was further stirred under atmospheric exposure for 12 h. The solvent was removed with a rotary evaporator and the residue was purified by column chromatography on silica gel, using ethyl acetate/hexane (1/50, *v*/*v*). A light yellow solid compound was obtained (0.615 g, 60%). ^1^H NMR (CDCl_3_): δ 8.17 (d, *J* = 6.6 Hz, 2H), 8.07 (d, *J* = 9.0 Hz, H), 7.80 (d, *J* = 8.4 Hz, H), 7.52 (t, *J* = 7.2 Hz, 2H), 7.45 (t, *J* = 7.8 Hz, H), 7.15 (d, *J* = 7.2 Hz, H), 6.98 (d, *J* = 8.4 Hz, H), 6.26 (s, H, NH), 3.25 (m, H, NCH), 2.99 (m, H), 2.90 (m, H), 2.09 (m, H), 1.91 (m, H), 1.84 (m, 2H, NCCH), 1.16 and 1.09 (d, *J* = 6.0 Hz, 6H, CH(C*H*_3_)_2_) ppm. ^13^C{^1^H} NMR (C_6_D_6_): δ 18.54, 18.92, 24.70, 26.86, 32.77, 56.80, 112.96, 116.71, 118.23, 126.85, 127.60, 128.95, 128.99, 129.07, 136.80, 137.74, 140.57, 141.68, 153.70 ppm. *m/z* calcd. ([M^+^] C_21_H_22_N_2_) 302.4210. Found: 302.1785.

### 2.8. Preparation of **10**

MeMgBr (1.24 mL, 3.11 M in diethyl ether) was added dropwise to a stirred suspension of HfCl_4_ (0.300 g, 0.938 mmol) in toluene (8 mL) at −78 °C. After stirring for 1 h at a controlled temperature within the range of −40 and −35 °C, the solution was cooled again to −78 °C. 2-Phenyl-1,2,3,4-tetrahydro-1,10-phenanthroline (0.24 g, 0.94 mmol) in toluene (4 mL) was added dropwise. The resulting solution was stirred at a controlled temperature within the range of −40 and −35 °C, for 2 h. Subsequently, it was stirred at room temperature overnight. The solvent was removed using a vacuum line and the residue was extracted with hexane (60 mL). The removal of the solvent produced a dark red solid compound, which was pure according to the results of the ^1^H and ^13^C NMR spectra analyses (Figure S10) (0.79 g, 53%). An analytical pure compound, containing single crystals that are suitable for X-ray crystallography, was obtained by recrystallization in hexane at −30 °C. ^1^H NMR (C_6_D_6_): δ 8.47 (d, *J* = 6.6 Hz, H), 7.63 (d, *J* = 7.8 Hz, H), 7.57 (d, *J* = 9.0 Hz, H), 7.47 (t, *J* = 6.0 Hz, H), 7.27 (dt, *J* = 7.8 Hz, H), 7.24 (d, *J* = 8.4 Hz, H), 7.07 (d, *J* = 7.2 Hz, H), 6.74 (d, *J* = 8.4 Hz, H), 3.95 (m, 2H), 2.62 (t, *J* = 6.6 Hz, 2H), 1.76 (m, 2H), 0.76 (s, 6H, Hf(CH_3_)_2_) ppm. ^13^C{^1^H} NMR (C_6_D_6_): δ 22.82, 26.84, 45.04, 63.72, 112.33, 115.03, 120.11, 124.29, 126.68, 128.62, 130.11, 130.33, 137.32, 141.01, 141.19, 147.77, 151.00, 163.28, 204.57 ppm. Anal. Calcd. (C_20_H_20_HfN_2_): C, 51.45; H, 4.32; N, 6.00%. Found: C, 51.35; H, 4.31; N, 6.10%.

### 2.9. Preparation of **11**

Complex **11** was prepared by the same procedure and experimental conditions as those employed for **10**, using 2-butyl-9-phenyl-1,2,3,4-tetrahydro-1,10-phenanthroline (0.390 g, 1.23 mmol). A dark red solid compound was obtained (0.148 g, 86%). An analytical pure compound, containing single crystals that are suitable for X-ray crystallography, was obtained by recrystallization in hexane at −30 °C. ^1^H NMR (C_6_D_6_): δ 8.49 (d, *J* = 6.6 Hz, H), 7.64 (d, *J* = 7.2 Hz, H), 7.60 (d, *J* = 9.0 Hz, H), 7.47 (t, *J* = 7.2 Hz, H), 7.27 (m, 2H), 7.14 (d, *J* = 8.4 Hz, H), 6.77 (d, *J* = 7.2 Hz, H), 4.29 (m, H, NCH), 2.83 (m, H), 2.52 (td, *J* = 4.2 Hz, H), 2.03 (m, H), 1.90 (m, H), 1.74 (m, 2H), 1.35 (m, 2H), 0.95 (t, *J* = 7.2 Hz, 3H), 0.85 (s, 3H, HfCH_3_), 0.79 (s, 3H, HfCH_3_) ppm. ^13^C{^1^H} NMR (C_6_D_6_): δ 14.48, 22.96, 23.20, 24.57, 28.82, 35.37, 53.14, 63.37, 65.49, 112.21, 115.01, 119.45, 124.21, 126.83, 128.49, 130.21, 130.30, 137.63, 141.24, 147.60, 150.43, 163.25, 204.42 ppm. Anal. Calcd. (C_24_H_28_HfN_2_): C, 55.12; H, 5.40; N, 5.36%. Found: C, 54.99; H, 5.41; N, 5.27%.

### 2.10. Preparation of **12**

Complex **12** was prepared by the same procedure and conditions as those employed for **10**, using 2-isopropyl-9-phenyl-1,2,3,4-tetrahydro-1,10-phenanthroline (0.20 g, 0.66 mmol). A dark red solid compound was obtained (0.330 g, 98%). An analytical pure compound, containing single crystals that are suitable for X-ray crystallography, was obtained by recrystallization in hexane at −30 °C. ^1^H NMR (C_6_D_6_): δ 8.50 (d, *J* = 7.2 Hz, H), 7.64 (d, *J* = 7.2 Hz, H), 7.60 (d, *J* = 9.0 Hz, H), 7.46 (t, *J* = 6.6 Hz, H), 7.26 (m, 2H), 7.10 (d, *J* = 7.8 Hz, H), 6.75 (d, *J* = 8.4 Hz, H), 4.00 (m, H, NCH), 2.76 (m, H), 2.58 (m, 2H), 1.84 (m, H), 1.68 (m, H), 1.74 (m, 2H), 1.05 and 1.01 (d, *J* = 7.2 Hz, 6H, CH(CH_3_)_2_), 0.88 (s, 3H, HfCH_3_), 0.78 (s, 3H, HfCH_3_) ppm. ^13^C{^1^H} NMR (C_6_D_6_): δ 18.61, 21.19, 23.12, 25.51, 32.68, 60.75, 63.82, 66.81, 112.40, 114.96, 120.50, 124.15, 126.91, 130.19, 130.31, 140.96, 141.37, 147.36, 150.96, 163.49, 204.30 ppm. Anal. Calcd. (C_23_H_26_HfN_2_): C, 54.28; H, 5.15; N, 5.50%. Found: C, 54.37; H, 5.01; N, 5.47%.

### 2.11. Preparation of **13**

*n*-BuLi (1.65 ml, 1.61 M in hexane) was slowly added to a stirred suspension of 2-naphthyl-1,10-phenanthroline (0.741 g, 2.42 mmol) in toluene (8 mL) at −10 °C. After stirring for 3 h at room temperature, degassed H_2_O (3 mL) was added. An aqueous layer was removed with a syringe under atmospheric N_2_. The solvent was removed using a vacuum line and the residue was dissolved in degassed ethanol (15 mL) and THF (5 mL). The solution was transferred to a bomb reactor, containing Pd/C (0.242 mmol, 10 mol %), under atmospheric N_2_. After the H_2_ gas was charged to 5 bar, it was stirred for 12 h at room temperature. The H_2_ gas was released and the catalyst residue was removed by filtration over Celite. The solvent was removed and the residue was purified by column chromatography on silica gel, using ethyl acetate/hexane (1/3, *v*/*v*). A light yellow solid compound was obtained (0.420 g, 47%). ^1^H NMR (C_6_D_6_): δ 8.49 (m, H), 7.75 (d, *J* = 8.4 Hz, H), 7.70 (m, H), 7.67 (d, *J* = 7.8 Hz, H), 7.64 (d, *J* = 7.2 Hz, H), 7.33 (m, 2H), 7.30 (m, 2H), 7.20 (d, *J* = 7.8 Hz, H), 7.02 (d, *J* = 8.4 Hz, H), 6.37 (s, H, NH), 3.16 (m, H, NCH), 2.82 (m, H), 2.73 (dt, *J* = 6.0 Hz, H), 1.79 (m, H), 1.57 (m, H), 1.27 (m, 2H), 1.12 (m, 4H), 0.77 (d, *J* = 7.2 Hz, 3H, CH_3_) ppm. ^13^C{^1^H} NMR (C_6_D_6_): δ 14.28, 23.11, 26.64, 27.95, 28.25, 36.44, 51.09, 112.87, 116.54, 122.68, 125.59, 126.08, 126.59, 126.65, 126.78, 128.69, 128.99, 129.39, 132.24, 134.64, 136.36, 137.68, 139.93, 141.57, 156.03 ppm. *m/z* calcd ([M^+^] C_26_H_26_N_2_) 366.5100. Found: 366.2094.

### 2.12. Preparation of **14**

Complex **14** was prepared by the same procedure and experimental conditions as those employed for **13**, using isopropyllithium (0.45 mL, 0.36 mmol, 0.79 M in pentane) and 2-naphthyl-1,10-phenanthroline (0.789 g, 2.58 mmol). A light yellow, sticky solid was obtained (0.388 g, 43%). ^1^H NMR (C_6_D_6_): δ 8.58 (d, *J* = 7.8 Hz, H), 7.75 (d, *J* = 9.0 Hz, H), 7.70 (d, *J* = 9.6 Hz, H), 7.66 (d, *J* = 7.2 Hz, H), 7.63 (d, *J* = 6.6 Hz, H), 7.32 (m, 4H), 7.18 (d, *J* = 8.4 Hz, H), 6.99 (d, *J* = 7.8 Hz, H), 6.39 (s, H, NH), 2.93 (m, H), 2.79 (m, H), 2.70 (dt, *J* = 4.8 Hz, H), 1.70 (m, H), 1.63 (m, H), 1.47 (m, H), 0.81 (d, *J* = 7.2 Hz, 3H, CH(CH_3_)_2_), 0.76 (d, *J* = 7.2 Hz, 3H, CH(CH_3_)_2_) ppm. ^13^C{^1^H} NMR (C_6_D_6_): δ 18.34, 18.77, 24.43, 26.78, 32.52, 56.73, 112.78, 116.67, 122.62, 125.59, 126.10, 126.51, 126.61, 126.86, 128.14, 128.69, 129.03, 129.28, 132.20, 134.71, 136.41, 137.64, 139.79, 141.75, 155.92 ppm. *m/z* calcd. ([M^+^] C_25_H_24_N_2_) 352.4800. Found: 352.1942.

### 2.13. Preparation of **15**

Complex **15** was prepared by the same procedure and experimental conditions as those employed for **10**, using **13** (0.366 g, 1.00 mmol). The product was sparingly soluble in hexane; therefore, it was extracted with toluene (50 mL). The trituration in hexane produced a dark brown powder (0.259 g, 45%). ^1^H NMR (C_6_D_6_): δ 8.65 (d, *J* = 7.2 Hz, H), 8.52 (d, *J* = 8.4 Hz, H), 7.95 (d, *J* = 9.0 Hz, H), 7.87 (d, *J* = 7.8 Hz, H), 7.77 (d, *J* = 8.4 Hz, H), 7.64 (d, *J* = 9.0 Hz, H), 7.41 (t, *J* = 7.8 Hz, H), 7.32 (t, *J* = 7.8 Hz, H), 7.18 (d, *J* = 8.4 Hz, H), 6.81 (d, *J* = 8.4 Hz, H), 4.33 (m, H), 2.88 (m, H), 2.57 (dt, *J* = 3.6 Hz, H), 2.11 (m, H), 1.92 (m, H), 1.79 (m, H), 1.38 (m, 4H), 0.96 (t, *J* = 6.6 Hz, 3H, (CH_2_) _3_CH_3_), 0.83 (s, 3H, HfCH_3_), 0.82 (s, 3H, HfCH_3_) ppm. ^13^C{^1^H} NMR (C_6_D_6_): δ 14.46, 23.20, 24.70, 28.82, 35.47, 53.35, 62.87, 65.21, 112.16, 119.18, 119.65, 124.47, 125.46, 126.69, 127.04, 129.64, 130.00, 130.22, 131.27, 133.32, 135.59, 140.81, 141.69, 144.07, 149.83, 164.16, 208.15 ppm. Anal. Calcd. (C_28_H_30_HfN_2_): C, 58.69; H, 5.28; N, 4.89%. Found: C, 58.79; H, 5.21; N, 4.87%.

### 2.14. Preparation of **16**

Complex **16** was prepared by the same procedure and experimental conditions as those employed for **10**, using **14** (0.303 g, 0.859 mmol). The product was sparingly soluble in hexane; therefore, it was extracted with toluene (50 mL). The trituration in hexane produced a dark brown powder (0.226 g, 47%). ^1^H NMR (C_6_D_6_): δ 8.66 (d, *J* = 7.8 Hz, H), 8.50 (d, *J* = 7.8 Hz, H), 7.92 (d, *J* = 9.0 Hz, H), 7.83 (d, *J* = 7.2 Hz, H), 7.76 (d, *J* = 8.4 Hz, H), 7.62 (d, *J* = 7.8 Hz, H), 7.40 (td, *J* = 7.2 Hz, H), 7.32 (m, H), 7.14 (d, *J* = 7.8 Hz, H), 6.77 (d, *J* = 7.2 Hz, H), 4.02 (m, H), 2.80 (m, H), 2.62 (dt, *J* = 6.0 Hz, H), 2.55 (m, H), 1.88 (m, H), 1.72 (m, H), 1.09 and 1.04 (d, *J* = 6.6 Hz, 6H, CH(CH_3_)_2_), 0.82 (s, 3H, HfCH_3_), 0.81 (s, 3H, HfCH_3_) ppm. ^13^C{^1^H} NMR (C_6_D_6_): δ 18.55, 21.28, 23.07, 25.44, 32.58, 60.98, 63.06, 66.88, 112.37, 119.64, 120.21, 124.55, 125.48, 126.81, 126.97, 129.31, 129.97, 130.26, 131.25, 133.82, 135.51, 140.97, 141.44, 143.94, 150.14, 164.58, 209.13 ppm. Anal. Calcd. (C_27_H_28_HfN_2_): C, 58.01; H, 5.05; N, 5.01%. Found: C, 57.91; H, 5.01; N, 5.11%.

### 2.15. Preparation of Anhydrous [(C_18_H_37_)_2_N(H)Me]^+^[B(C_6_F_5_)_4_]^−^

[(C_18_H_37_)_2_N(H)Me]^+^[B(C_6_F_5_)_4_]^−^, which was prepared according to the method reported in patent, contained water, [36] which caused some problems in the activation reactions. The water contained in [(C_18_H_37_)_2_N(H)Me]^+^[B(C_6_F_5_)_4_]^−^ was not removed by the conventional ways (i.e., evacuation at 60 °C, refluxing with the Dean-Stark apparatus after dissolving in toluene, or treatment with molecular sieves in methylcyclohexane). The ^19^F NMR spectrum indicated that the K^+^[B(C_6_F_5_)_4_]^−^ that was purchased from Alfa Aesar, contained 10 mol % impurity. Therefore, excess K^+^[B(C_6_F_5_)_4_]^−^ (0.633 g, 0.881 mmol, based on the assumption that it is pure) was reacted with [(C_18_H_37_)_2_N(H)Me]^+^[Cl]^−^ (0.404 g, 0.705 mmol) in toluene (anhydrous, 10 mL), for 1 h, at room temperature, inside a glove box. After filtration over Celite, the solvent was removed using a vacuum line. The residue was dissolved in methylcyclohexane (4 mL) and filtered again over Celite. The removal of the solvent produced a yellow oily compound, which was used without further purification (0.797 g, 93 %). In the ^1^H NMR spectrum of the water-containing [(C_18_H_37_)_2_N(H)Me]^+^[B(C_6_F_5_)_4_]^−^, prepared according to the patent method, NC*H*_2_ protons were observed as a single broad signal around 1.89 ppm (Figure S28). In contrast, in the ^1^H NMR spectrum of anhydrous [(C_18_H_37_)_2_N(H)Me]^+^[B(C_6_F_5_)_4_]^−^, the two protons attached on the α-carbon (NC*H*_2_) are separately observed at 1.97 and 1.80 ppm (Figure S27). ^1^H NMR (C_6_D_6_): δ 3.15 (br, H, NH), 1.97 (m, 2H, NCH_2_), 1.80 (m, H, NCH_2_), 1.51 (d, *J* = 6.0 Hz, 3H, NCH_3_), 1.45–1.29 (m, 48H), 1.26 (quintet, *J* = 7.2 Hz, 4H), 1.13 (quintet, *J* = 7.2 Hz, 4H), 0.94 (t, *J* = 7.8 Hz, 6H), 0.88 (quintet, *J* = 7.8 Hz, 4H), 0.81 (m, 4H) ppm. ^19^F NMR (C_6_D_6_): δ −132.09, −161.75, −165.98.

### 2.16. A Typical Polymerization (Entry 8 in Table 1)

A bomb reactor (125 mL) was evacuated at 60 °C for 1 h. After charging with ethylene gas at atmospheric pressure, a solution of Me_3_Al (28.8 mg, 200 µmol-Al) in methylcyclohexane (15.5 g) was added to the reactor. The mixture was stirred for 1 h at 100 °C using a mantle, and the solution was subsequently removed using a cannula. The reactor was evacuated once more to remove any residual solvent and was re-charged with ethylene gas at atmospheric pressure. This procedure was performed to clean up any catalyst poisons. The reactor was charged with methylcyclohexane (15.5 g), which contains MMAO (AkzoNobel, 6.7 wt %-Al in heptane, 20 mg, 50 µmol-Al) and the temperature was set to 80 °C. A solution of (octyl)_2_Zn (100 µmol) in methylcyclohexane (10.0 g) was charged. Subsequently, the methylcyclohexane solution (0.30 g) containing the catalyst **12** (2.0 µmol-Hf) that was activated with [(C_18_H_37_)_2_N(H)Me]^+^[B(C_6_F_5_)_4_]^−^ (1.0 eq) in benzene, was injected. Ethylene/propylene mixed gas (10 bar/10 bar, total 20 bar) was charged from a tank into the reactor at 20 bar, and the polymerization was performed for 50 min. The temperature was controlled at 80–90 °C. The remaining ethylene/propylene mixed gas was vented off and the reactor was cooled to 75 °C. The generated polymer was collected and dried in a vacuum oven at 160 °C overnight to obtain 6.3 g of the polymer.

### 2.17. X-ray Crystallography

The reflection data for **1**, **3**, **6**, **10**, **11**, and **12** were collected on a Bruker APEX II CCD area diffractometer (Billerica, MA, USA), using graphite-monochromated Mo K–α radiation (λ = 0.7107 Å). Specimens of suitable quality and sizes were selected, mounted, and centered in the X-ray beam using a video camera. The hemisphere of the reflection data was collected as φ and ω scan frames at 0.5°/frame and an exposure time of 10 s/frame. The cell parameters were determined and refined by the SMART program. Data reduction was performed using the SAINT software (Madison, WI, US). The data were corrected for Lorentz and polarization effects. An empirical absorption correction was applied using the SADABS program. The structures of the compounds were determined by direct methods and refined by the full matrix least-squares methods, using the SHELXTL program package with anisotropic thermal parameters for all non-hydrogen atoms. CCDC 1918314–1918319 contain the supplementary crystallographic data for this paper. These data can be obtained free of charge via http://www.ccdc.cam.ac.uk/conts/retrieving.html (or from the CCDC 12 Union Road, Cambridge CB2 1EZ, UK; Fax: +44 1223 336033; E-mail: deposit@ccdc.cam.ac.uk). Crystallographic data for **1** (CCDC# 1918314): C_20_H_27_HfN, *M* = 459.91, monoclinic, *a* = 7.7840(2), *b* = 31.3344(6), *c* = 7.7852(2) Å, β = 104.9576(15)°, *V* = 1834.52(8) Å^3^, *T* = 100(2) K, space group *P2_1_/n*, *Z* = 4, 3395 unique (R(int) = 0.1398), which were used in all the calculations. The final *wR*_2_ was 0.0831 (*I* > 2σ(*I*)). Data for **3** (CCDC# 1918315): C_21_H_25_HfNS, *M* = 501.97, monoclinic, *a* = 10.7974(2), *b* = 11.5868(2), *c* = 15.5040(3) Å, β = 93.1287(9)°, *V* = 1936.77(6) Å^3^, *T* = 296 K, space group *P2_1_/c*, *Z* = 4, 3563 unique (R(int) = 0.0350), which were used in all calculations. The final *wR*_2_ was 0.0349 (*I* > 2σ(*I*)). Data for **6** (CCDC# 1918316): C_25_H_25_HfN, *M* = 517.95, monoclinic, *a* = 8.7930(2), *b* = 14.7370(3), *c* = 15.7492(3)Å, β = 92.1487(10)°, *V* = 2039.38(7) Å^3^, *T* = 100(2) K, space group *P2_1_/c*, *Z* = 4, 4256 unique (R(int) = 0.0327), which were used in all the calculations. The final *wR*_2_ was 0.0496 (*I* > 2σ(*I*)). Data for **10** (CCDC# 1918317): C_20_H_18_HfN_2_, *M* = 464.85, orthorhombic, *a* = 12.5672(8), *b* = 7.8737(5), *c* = 34.8321(19) Å, *V* = 3446.6(4) Å^3^, *T* = 100(2) K, space group *Pbca*, *Z* = 8, 1002 unique (R(int) = 0.2063), which were used in all the calculations. The final *wR*_2_ was 0.0807 (*I* > 2σ(*I*)). Data for **11** (CCDC# 1918319): C_24.70_H_28.60_HfN_2_, *M* = 531.98, monoclinic, *a* = 16.1724(3), *b* = 16.1772(3), *c* = 22.6003(4)Å, β = 107.9164(12)°, *V* = 5626.05(18) Å^3^, *T* = 100(2) K, space group *P2_1_*, *Z* = 10, 20485 unique (R(int) = 0.1342), which were used in all the calculations. The final *wR*_2_ was 0.1062 (*I* > 2σ(*I*)). Data for **12** (CCDC# 1918318): C_23_H_26.20_HfN_2_, *M* = 509.15, orthorhombic, *a* = 16.2081(7), *b* = 24.608(1), *c* = 27.4705(12) Å, *V* = 10956.6(8) Å^3^, *T* = 100(2) K, space group *P2_1_2_1_2_1_*, *Z* = 20, 7082 unique (R(int) = 0.1888), which were used in all calculations. The final *wR*_2_ was 0.1383 (*I* > 2σ(*I*)).

## 3. Results and Discussion

### 3.1. Preparation of Hf Complexes

**Scheme 2:**
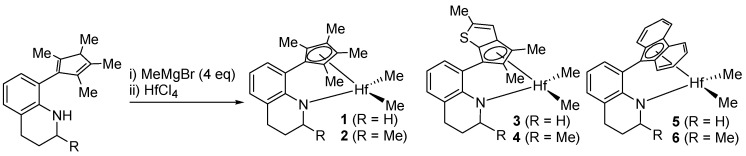
Synthesis of Hf-based half-metallocene complexes.

A series of Ti- and Zr-based half-metallocene complexes [*ortho*-C_6_H_4_(L)(NR)]MMe_2_ (M = Ti or Zr) were prepared for ethylene/α-olefin copolymerizations. It was found that the Ti-complexes with L being tetramethylcyclopentadienyl or thiophene-fused dimethylcyclopentadienyl and R being linked to *ortho*-phenylene bridge exhibited excellent catalytic performance [33,34,37,38]. Titanium complexes were prepared on a large scale simply by treating the ligand precursor successively with 4 equiv MeMgCl and TiCl_4_·(DME) (DME = dimethoxyethane). Attempts to synthesize the Hf-analogues by the same procedure (i.e., treatment of the ligand precursor successively with four equiv MeMgCl and HfCl_4_·(THF)_2_ were unsuccessful [39]. The syntheses of Ti- and Zr-based half-metallocene complexes have been widely reported. However, the syntheses of Hf-analogues are seldom reported [40]. [Me_2_Si(η^5^-Me_4_C_5_)(N*^t^*Bu)]HfCl_2_ was obtained in a rather low yield (38%) by reacting [Me_2_Si(η^5^-Me_4_C_5_)(N*^t^*Bu)]Li_2_ with HfCl_4_·(THF)_2_, whereas the corresponding reaction with ZrCl_4_·(THF)_2_ afforded the desired complex [Me_2_Si(η^5^-Me_4_C_5_)(N*^t^*Bu)]ZrCl_2_ in high yield (74%) [41,42]. Eventually, we found that some commercial sources of HfCl_4_ contained water, which was the cause of the failure. The use of sublimed-grade of HfCl_4_ instead of its water-containing counterpart, and MeMgBr instead of MeMgCl, cleanly afforded the desired half-metallocene Hf complexes in good yield (84 %) (Scheme 2). Along with tetrahydroquinoline- and tetrahydroquinaldine-linked tetramethylcyclopentadienyl HfMe_2_ complexes (**1** and **2**), and tetrahydroquinoline- and tetrahydroquinaldine-linked thiophene-fused dimethylcyclopentadienyl HfMe_2_ complexes (**3** and **4**), of which Ti analogues were reported to show excellent performance, fluorenyl congeners (**5** and **6**) were also prepared because the excellent performance of [Me_2_Si(η^5^-fluorenyl)(NR)]TiMe_2_ has also been reported [43]. The ^1^H NMR and ^13^C NMR spectra were in agreement with the expected structures (Figures S1–S6) and the structures of **1**, **3**, and **6** were confirmed by X-ray crystallography.

**Scheme 3:**
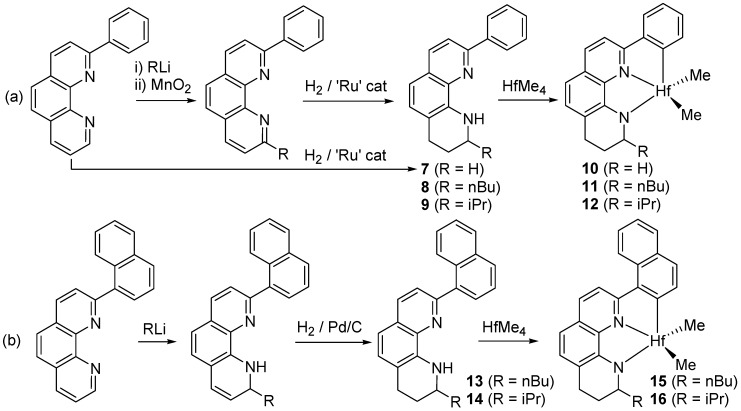
Synthesis of [N^amido^,N,C^aryl^]HfMe_2_-type post-metallocene complexes bearing phenyl moiety (**a**) and naphthyl moiety (**b**).

**Figure 1:**
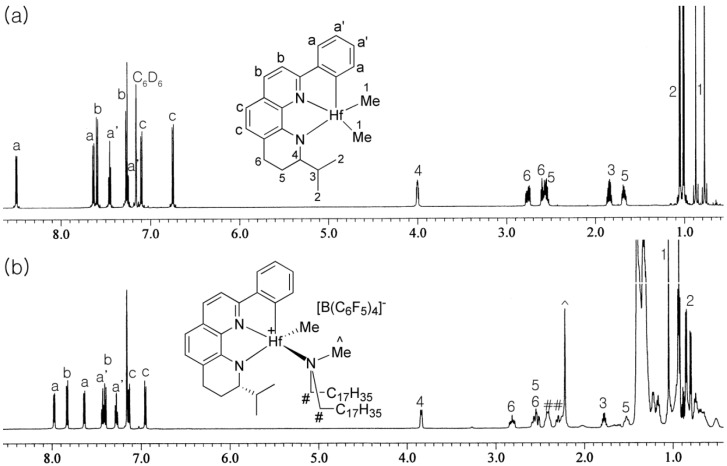
^1^H NMR spectra of **12** (**a**) and its activated complex (**b**).

Pyridylamido-Hf complex **III** is a [N^amido^,N^pyridine^,C^aryl^]HfMe_2_-type complex, which contains characteristic Hf-C(aryl) bonds [44]. Through olefin insertion into the Hf-C(aryl) bond at the initial stage of polymerization, the ligand structure was modified and accordingly, the coordination geometry changed significantly [9,45]. Polymer chains are grown from the active species with a modified ligand structure. Namely, the Hf-C(aryl) bond plays a critical role in the high performance; we attempted, in this work, to prepare similar [N^amido^,N,C^aryl^]HfMe_2_-type complexes containing the Hf-C(aryl) bond, with tetrahydrophenanthroline framework (Scheme 3). 9-Phenyl-1,2,3,4-tetrahydro [1,10] phenanthroline (**7**) and 2-butyl-9-phenyl-1,2,3,4-tetrahydro[1,10]phenanthroline (**8**) were known compounds and 2-isopropyl-9-phenyl-1,2,3,4-tetrahydro[1,10]phenanthroline (**9**) was synthesized by the modification of the reported method and conditions (Scheme 3a) [35]. The treatment of **7**–**9** with HfMe_4_, generated in situ by the reaction of four equiv MeMgBr and HfCl_4_, afforded the targeted [N^amido^,N,C^aryl^]HfMe_2_-type complexes, which contain a Hf-C(aryl) bond [46]. HfMe_4_ is unstable; therefore, it should be generated and reacted in situ at a low temperature (−35 to −40 °C). The Hf-C(aryl) bond formation was evident from the result of the ^1^H NMR spectrum analysis (Figure 1a). The *ortho*-metalated phenylene (N=C-C_6_H_4_-Hf) signals were clearly observed at 8.50 (d), 7.84 (t), 7.46 (t), and 7.26 (d) ppm with integration value ratios of 1:1:1:1 in the ^1^H NMR spectrum, whereas phenyl (–C_6_H_5_) signals were observed at 8.18 (d), 7.52 (t), and 7.45 (t) ppm with integration value ratios of 2:2:1 in those of ligand precursors **7**–**9**. The structures of **10**–**12** were unambiguously confirmed by X-ray crystallography.

Analogues ligand precursors containing a naphthyl substituent instead of phenyl (**13** and **14** in Scheme 3b) were not synthesized according to the same synthetic scheme, because naphthyl group was also involved in the hydrogenation process. **13** and **14** were successfully synthesized through the selective hydrogenation of the intermediates that were captured from the reaction of 2-naphthylphenanthroline with RLi. To avoid the hydrogenation of the naphthyl group, the Pd/C catalyst was used instead of the Ru-complex and H_2_ pressure was lowered to 5 bar. From **13** and **14**, the targeted complexes (**15** and **16**) were successfully prepared by the treatment of the in situ generated HfMe_4_. The ^1^H and ^13^C NMR spectra were in agreement with the expected structure (Figures S13 and S14).

### 3.2. X-ray Crystallographic Studies

**Figure 2:**
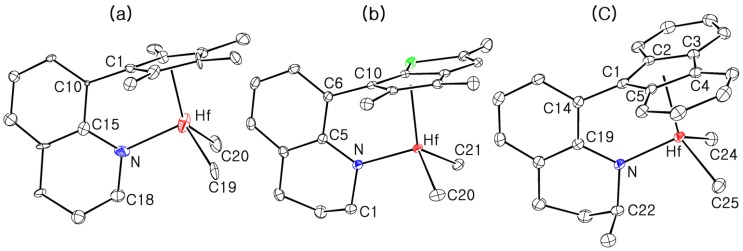
Thermal ellipsoid plot (30% probability level) of **1** (**a**), **3** (**b**), and **6** (**c**). Hydrogen atoms are omitted for clarity. Selected bond distances (Å) and angles (°) in **1** (**a**): Hf-N, 2.15(2): Hf-C(19), 2.20(2); Hf-C(20), 2.36(2); C_5_(centroid)-Hf, 2.01; C_5_(centroid)-Hf-N, 102.2; C(19)-Hf-C(20), 101.9(9); C_5_(centroid)-C(1)-C(10), 170.1; C(1)-C_5_(centroid)-Hf, 91.6; C(15)-N-Hf, 124.3(1); C(18)-N-Hf, 121.2(1); C(15)-N-C(18), 114.3(2). In **3** (**b**): Hf-N, 2.038(2): Hf-C(20), 2.217(3); Hf-C(21), 2.223(3); C_5_(centroid)-Hf, 2.17; C_5_(centroid)-Hf-N, 102.1; C(20)-Hf-C(21), 103.7(1); C_5_(centroid)-C(10)-C(6), 171.1; C(10)-C_5_(centroid)-Hf, 88.2; C(1)-N-Hf, 117.0(2); C(5)-N-Hf, 126.9(2); C(1)-N-C(5), 116.1(2). In **6** (**c**): Hf-N, 2.022(3): Hf-C(24), 2.227(4); Hf-C(25), 2.510(3); C_5_(centroid)-Hf, 2.22; C(1)-Hf, 2.451(3); C(2)-Hf, 2.505(3); C(5)-Hf, 2.510(3); C(3)-Hf, 2.540(3); C(4)-Hf, 2.541(3); C_5_(centroid)-Hf-N, 102.4; C(24)-Hf-C(25), 103.7(1); C_5_(centroid)-C(1)-C(14), 172.5; C(1)-C_5_(centroid)-Hf, 86.7; C(19)-N-Hf, 126.5(2); C(22)-N-Hf, 118.8(2); C(19)-N-C(22), 114.5(2).

The molecular structures of the half-metallocene Hf complexes of **1**, **3**, and **6** were confirmed by X-ray crystallography (Figure 2). The sum of the bond angles around the N atom is in all cases 360°, indicating that the N atoms adopt a sp^2^-hybridization for the π-donation from N to the Hf atom. The Hf-N distances are in the order of **1** > **3** > **6** (2.150(2), 2.038(2), and 2.022(3) Å, respectively) and these are substantially longer than the Ti-N distances observed for the corresponding Ti complexes (1.929(2), 1.936(3), and 1.921(2) Å, respectively) [33,34]. The C_5_(centroid)-Hf distances are reversely in the order of **6** > **3** > **1**, and the observed distance for **1** (2.01 Å), is similar to the C_5_(centroid)-Ti distance observed for the corresponding Ti complex (2.02 Å), while those for **3** and **6** (2.17 and 2.22 Å) are longer than the C_5_(centroid)-Ti distances observed for the corresponding Ti complexes (2.03 and 2.07 Å). The C_5_(centroid)-Hf-N angles (102.2°, 102.1°, and 102.4° for **1**, **3**, and **6**, respectively) are substantially more acute than the C_5_(centroid)-Ti-N angles observed for the corresponding Ti complexes (106.1°, 106.8°, and 106.9°). The C_5_(centroid)-Ti-N angles have been used as a qualitative measure for the constrained geometry. The more acute the angle, the more open the reaction site becomes.

**Figure 3:**
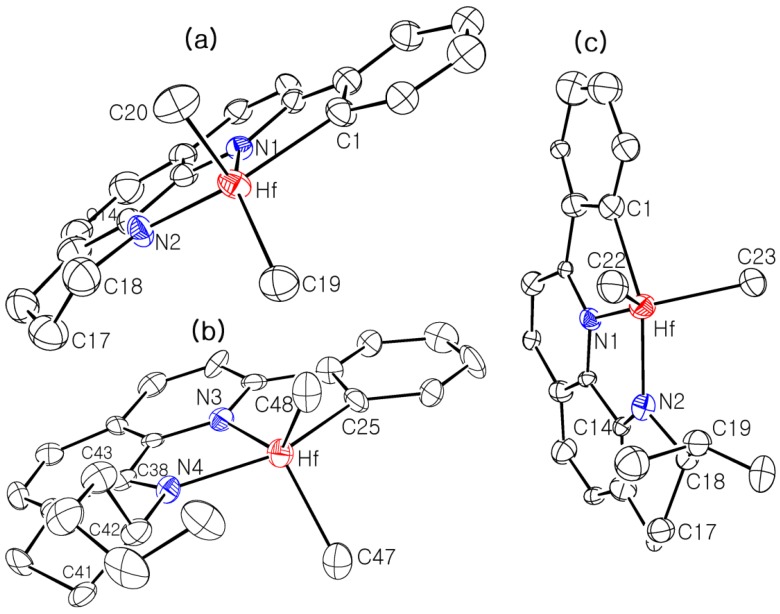
Thermal ellipsoid plot (30% probability level) of **10** (**a**), **11** (**b**), and **12** (**c**). Hydrogen atoms are omitted for clarity. Selected bond distances (Å) and angles (°) in **10** (**a**): Hf-N(1), 2.30(2); Hf-N(2), 2.11(2); Hf-C(1), 2.27(2); Hf-C(19), 2.22(2); Hf-C(20), 2.22(2); N(1)-Hf-C(19), 137.4(7); N(1)-Hf-C(20), 118.9(7); C(19)-Hf-C(20), 103.6(9); N(2)-Hf-C(1), 140.4(8); C(14)-N(2)-Hf, 124.2(1); C(18)-N(2)-Hf, 121.3(2); C(14)-N(4)-C(18), 114.5(2); Hf-N(2)-C(18)-C(17), 29.2. In **11** (**b**): Hf-N(3), 2.30(2); Hf-N(4), 2.12(2); Hf-C(25), 2.25(2); Hf-C(47), 2.20(2); Hf-C(48), 2.26(2); N(3)-Hf-C(48), 133.5(8); N(3)-Hf-C(47), 124.2(8); C(47)-Hf-C(48), 102.1(1); N(4)-Hf-C(25), 139.7(9); C(38)-N(4)-Hf, 122.0(1); C(42)-N(4)-Hf, 121.2(2); C(38)-N(4)-C(42), 116.6(2); Hf-N(4)-C(42)-C(43), 91.1; Hf-N(4)-C(42)-C(41), 38.4. In **12** (**c**): Hf-N(1), 2.23(3); Hf-N(2), 2.14(3); Hf-C(1), 2.39(5); Hf-C(22), 2.28(5); Hf-C(23), 2.27(5); N(1)-Hf-C(22), 136.1(2); N(1)-Hf-C(23), 121.6(2); C(22)-Hf-C(23), 101.5(2); N(2)-Hf-C(1), 141.1(2); C(14)-N(2)-Hf, 122; C(18)-N(2)-Hf, 120; C(14)-N(2)-C(18), 115; Hf-N(2)-C(18)-C(19), 33; Hf-N(2)-C(18)-C(17), 17.

The molecular structures of the post-metallocene Hf complexes of **10**, **11**, and **12** were also confirmed by X-ray crystallography (Figure 3). Despite the unsatisfactory quality of the data, the molecular structures can be seen clearly. The coordination geometry can be defined as a distorted trigonal bipyramid; N(pyridine), C(methyl), C(methyl), and Hf atoms form a plane (sum of the bond angles around Hf atoms, 360°), while C(aryl) and N(amido) atoms distortedly occupy the axial sites (C(aryl)-Hf-N(amido) angles, 140°). The sum of the bond angles around the N(amido) atoms in **10, 11**, and **12** are perfectly 360° or close to 360° (360°, 360°, and 357°, respectively), indicating that the N atoms adopt an sp^2^-hybridization for the π-donation from N to the Hf atom. All the atoms in the ligand framework except a CH_2_ (i.e., N-C(R)H***CH***_2_) fragment, are situated nearly in a plane with the Hf atom, while the C(methyl)-Hf-C(methyl) plane perpendicularly bisects the plane of the ligand framework (the angle between the two planes, 87°). The butyl group in **11** is directed nearly perpendicularly from the plane of the ligand framework (Hf-N(4)-C(42)-C(43) dihedral angle, 91°), while the isopropyl group in **12** is askew form the plane (Hf-N(2)-C(18)-C(19) dihedral angle, 33°). The Hf-C(methyl) distances are in the order of **12** (2.28(5), 2.27(5) Å) > **11** (2.20(2), 2.26(2) Å) > **10** (2.22(2), 2.22(2) Å).

### 3.3. Activation Reactions

The activation reaction of the prototype pyridylamidohafnium complex **III** is rather tricky and complex [47,48]. The reaction with B(C_6_F_5_)_3_ results in an activated complex; however, the generated complex was decomposed through a process involving C_6_F_5_ transfers. The reaction with [Ph_3_C]^+^[B(C_6_F_5_)_4_]^−^ may immediately afford the targeted ion-pair complex {[N,N,C^naphthyl^]HfMe}^+^[B(C_6_F_5_)_4_]^−^, which also decomposes especially when exposed to sunlight or a polar solvent. The reaction with [PhN(H)Me_2_]^+^[B(C_6_F_5_)_4_]^−^ results in protonation on the Hf-C^Naphthyl^ bond to generate a {[N,N]HfMe_2_}^+^[B(C_6_F_5_)_4_]^−^-type complex, which further reacts with the generated byproduct (PhNMe_2_) to produce an undesired complex. The best activator was the aliphatic amine-based ammonium salt, [(C_18_H_37_)_2_N(H)Me]^+^[B(C_6_F_5_)_4_]^−^, which cleanly afforded the desired ion-pair complex, {[N,N,C^naphthyl^]HfMe}^+^[B(C_6_F_5_)_4_]^−^. The activated ion-pair complex is stable in benzene [18].

**Scheme 4:**
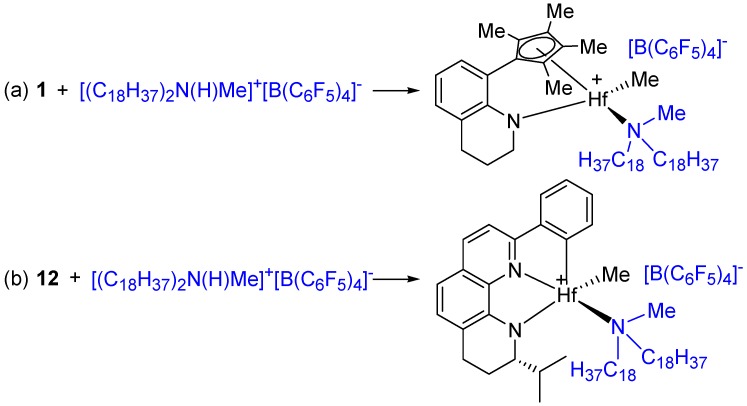
Activation reaction of **1** (**a**) and **12** (**b**) with [(C_18_H_37_)_2_N(H)Me]^+^[B(C_6_F_5_)_4_]^−^.

When the half-metallocene complex **1** was reacted with [(C_18_H_37_)_2_N(H)Me]^+^[B(C_6_F_5_)_4_]^−^ in C_6_D_6_, a single set of signals was observed in the ^1^H NMR spectrum (Figure S15), which was assigned to the desired ion-pair complex generated by the protonation on Hf-Me (Scheme 4a). The reaction was rather slow, requiring several hours, and the generated ion-pair complex was stable in C_6_D_6_. (C*H*_3_)_4_C_5_ signals were separately observed at 2.02, 1.92, 1.70, and 1.58 ppm as sharp singlets, which are indicative of the tight binding of (C_18_H_37_)_2_NMe to a vacant site on the Hf center that was generated by the methide abstraction. At the structural point of amine tight binding, the Hf center becomes a chiral center; the two α-methylene carbons and furthermore, the two protons attached on each α-methylene carbon on (C_18_H_37_)_2_NMe, are inequivalent and NC*H*_2_ resonances were separately observed at 2.34, 2.26, and 2.18 ppm. In the ^19^F NMR spectrum, signals assignable to *ortho*-, *meta*-, and *para*-fluorine of –C_6_F_5_ were observed. The analyses of the ^1^H NMR spectra of the complexes generated by the action of [(C_18_H_37_)_2_N(H)Me]^+^[B(C_6_F_5_)_4_]^−^ to **2**–**6** indicated that the desired ion-pair complexes were cleanly generated (Figures S16–S20). 

When **12**, bearing the isopropyl substituent, was reacted with [(C_18_H_37_)_2_N(H)Me]^+^[B(C_6_F_5_)_4_]^−^ in C_6_D_6_, the desired ion-pair complex was immediately generated with the concomitant generation of methane (Scheme 4b). The generated complex was stable in C_6_D_6_ for several days. A single set of signals assignable to the desired ion-pair complex was observed in the ^1^H NMR spectrum (Figure 1b). The Hf-C*H*_3_ signal was observed at 1.06 ppm as a singlet. Amine (C_18_H_37_)_2_NMe seems to bind to the Hf center rather loosely. The NC*H*_2_ and NC*H*_3_ signals are relatively broad compared with those observed for the activated complexes of **1**–**6**. The analyses of the ^1^H NMR spectra recorded on the activation reactions of **10** and **11** indicated that the desired ion-pair complexes were also generated by the action of [(C_18_H_37_)_2_N(H)Me]^+^[B(C_6_F_5_)_4_]^−^ (Figures S21 and S22). However, in those cases, the NC*H*_2_ and NC*H*_3_ signals are much broader than in the case of **12**, although the signals assigned to the ligand framework and Hf-C*H*_3_ are similarly sharp. For **15** and **16**, which bear naphthyl groups, some solid was precipitated when they were reacted with [(C_18_H_37_)_2_N(H)Me]^+^[B(C_6_F_5_)_4_]^−^ in C_6_D_6_. However, the analyses of the ^1^H NMR spectra of the soluble portion indicated the generation of the desired ion-pair complexes, although the yield was low (~70%) (Figures S24 and S25).

### 3.4. Polymerization Studies

The prepared complexes, which were (**1**–**6**, **10**–**12**, and **15**,**16**) activated with [(C_18_H_37_)_2_N(H) Me]^+^[B(C_6_F_5_)_4_]^−^, were screened for ethylene/propylene copolymerization in methylcyclohexane, at an initial temperature of 80 °C, under 20 bar of ethylene/propylene mixed gas. Half-metallocene hafnium complexes **1**–**6** were inactive, although the activation reaction with [(C_18_H_37_)_2_N(H) Me]^+^[B(C_6_F_5_)_4_]^−^ cleanly generated the desired ion-pair complexes. Complexes **10**, **11**, and **12** were active and their activity was increased as the increase in the steric bulkiness of the attached substituent and the highest activity was observed with **12** bearing an isopropyl substituent (entry 3 in Table 1). However, it did not compete with the prototype Dow catalyst **III**; the productivity of **12** was ~half of **III** (entry 3 vs. 5). For the prototype catalysts, **III**, which bears a naphthyl group, exhibited higher activity than its analogue, bearing a phenyl substituent. However, in our case, replacing the phenyl group with naphthyl resulted in lowered activity (i.e., **12** vs. **16**; entry 3 vs. 4). The Hf-C bonding is significantly ionic and the bonding energy may be sensitive to the steric congestion [49]. When the steric congestion is insignificant, the ionic Hf-C bond becomes strong and the olefin insertion through it may be less favorable, leading to lowered activity. We hypothesized that the Hf center in **12** is not as sterically congested as it is in **III**. Typically, the steric hindrance around the metal center influences the comonomer incorporation; the more widely opened the reaction site, the higher the incorporation of α-olefin. However, the prototype Dow catalyst **III** is exceptional in the incorporation of high amount of α-olefin, regardless of the sterical congestion of the reaction site. Whereas **III** was able to incorporate 56 mol % of propylene, **10**–**12** and **16** incorporated only 10–13 mol % of propylene under the same reaction condition. Accordingly, the polymers generated by **III** are amorphous, while the polymers generated by **10**–**12** and **16** exhibited melting signals around 100 °C. We also reported various type of Hf complexes ([N,P]Hf(CH_2_Ph)_3_, [N,P,N]HfMe_2_, and [N,N]Hf(CH_2_Ph)_3_-type) with tetrahydroquinoline and tetrahydrophenanthroline frameworks, which were also inferior to **III** in terms of the α-olefin incorporation capability as well as the activity [50,51,52].

**Figure 4:**
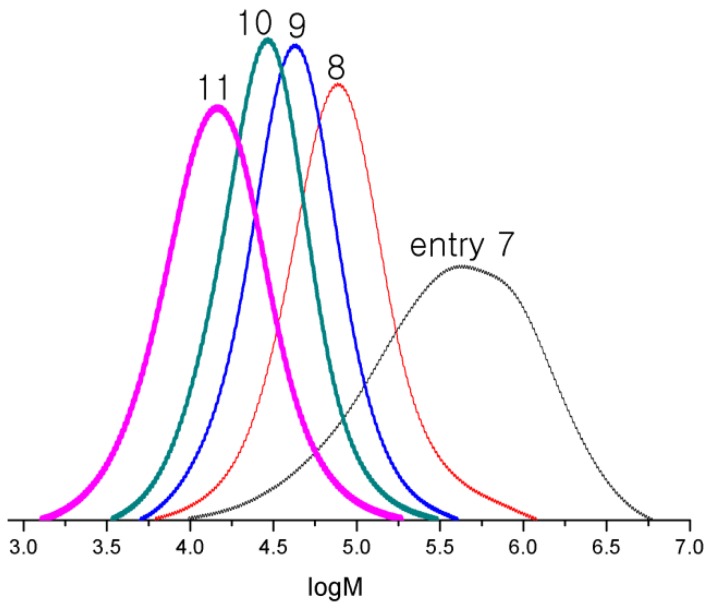
GPC curves for the polymers obtained in CCTP.

A higher molecular weight polymer was generated with **12** relative to **III** (*M*_n_, 124 kDa vs. 61 kDa). The *M*_n_ value of the generated polymer was increased further to 190 kDa by replacing trioctylaluminum with MMAO, which was employed as a scavenger. These results indicated that trioctylaluminum was engaged in the chain transfer process through the alkyl exchange between Al and the chain-growing Hf centers, leading to a lowered molecular weight, and that such chain transfer reactions could be suppressed by employing MMAO instead of trioctylaluminum as a scavenger. The unique advantage of **III** over the other types of catalysts is that it can be used in CCTP, which is performed in the presence of chain transfer agent (R_2_Zn) deliberately added. **III** is capable of generating PO chains attached on Zn sites (i.e., (polyolefinyl)_2_Zn) with negligible a β-elimination process, which process is inevitable with the conventional metallocene and half-metallocene catalysts. When the polymerizations were performed with **12** in the presence of (octyl)_2_Zn, which was deliberately added as a chain transfer agent, the *M*_n_ values were sensitive to the amount of (octyl)_2_Zn and the observed *M*_n_ values after the universal calibration (i.e., converted by the equation ‘*M*_PO_ = 0.495 × *M*_PS_^0.990^/(1 – S)’, where S is the mass fraction of the CH_3_–side chains, i.e., S = (15 × [C_3_H_6_])/[(1 – [C_3_H_6_]) × 28 + ([C_3_H_6_] × 42)]) [11] were in good agreement with the expected values, calculated based on the amount of (octyl)_2_Zn employed and the amount of generated polymer (*M*_n_^expected^ = yield (g)/(2 × Zn-mol))·(*M*_n_^PO-equivalent^ = 30, 17, 12, 5.9 kDa vs. *M*_n_^expected^ = 32, 17, 9.0, 4.5, respectively; entries 8–11; Figure 4). These results indicated that **12** worked well in CCTP, successfully converting the (octyl)_2_Zn to (polyolefinyl)_2_Zn with a negligible β-elimination process, although the activity and the capability for α-olefin incorporation were inferior compared to those of **III**.

## 4. Conclusions

A series of half metallocene HfMe_2_ complexes bearing a tetrahydroquinoline or tetrahydroquinaldine framework and tetramethylcyclopentadienyl, thiophene-fused dimethylcyclopentadienyl, or fluorenyl ligand, were prepared by sequential treatments of four equiv. MeMgBr and HfCl_4_ to the ligand precursors. A series of [N^amido^,N,C^aryl^]HfMe_2_-type post-metallocene complexes bearing a tetrahydrophenanthroline framework, substituted with n-butyl or isopropyl group at position 2 and phenyl or naphthyl group at position 9, were prepared by the treatment of the ligand precursor with in situ generated HfMe_4_. The structures of many of the prepared complexes were confirmed by X-ray crystallography. The activation reaction of the half metallocene HfMe_2_ complexes with [(C_18_H_37_)_2_N(H)Me]^+^[B(C_6_F_5_)_4_]^−^ cleanly afforded the desired ion-pair complexes, although the reaction was slow. However, the activated complexes were inactive in ethylene/propylene copolymerization. The activated complexes, generated from post-metallocene HfMe_2_ complexes by the action of [(C_18_H_37_)_2_N(H)Me)]^+^[B(C_6_F_5_)_4_]^−^ were active. The highest activity was observed with **12**, which bears a bulky isopropyl group. However, **12** was inferior to the prototype pyridylamido-Hf Dow catalyst (**III**) in terms of the activity and α-olefin incorporation capability. Furthermore, **12** performed well in the CCTP, which was performed in the presence of (octyl)_2_Zn, converting the (octyl)_2_Zn to (polyolefinyl)_2_Zn with controlled lengths of the polyolefinyl chain.

## Figures and Tables

**Table 1:** Polymerization results ^a^.

Entry	Catalyst	Al ^b^ (50 µmol)	Zn ^c^ (μmol)	Yield (g)	[C_3_H_6_] ^d^ (mol%)	*T*_m_ (°C)	*M*_n_^e^ (kDa)	*M*_w_/*M*_n_
1	**10**	TOA	0	1.2	10	86–129	16	11.0
2	**11**	TOA	0	5.8	12	101–120	87	1.5
3	**12**	TOA	0	7.5	13	102–120	124	3.2
4	**16**	TOA	0	3.5	11	99–115	26	2.0
5	**III**	TOA	0	16	56	not-detected	61	2.6
6	**11**	MMAO	0	5.5	10	105–120	266	2.1
7	**12**	MMAO	0	7.8	11	102–117	190	3.4
8	**12**	MMAO	100	6.3	9.0	100–117	58 (30; 32) ^f^	1.8
9	**12**	MMAO	200	6.6	11	100–112	33 (17; 17) ^f^	1.6
10	**12**	MMAO	300	5.4	13	97–110	22 (12; 9.0) ^f^	1.6
11	**12**	MMAO	400	3.6	15	94–108	11 (5.9; 4.5) ^f^	1.8

^a^ Polymerization conditions: Hf complex (2.0 μmol), activator ([(C_18_H_37_)_2_N(H)Me]^+^[B(C_6_F_5_)_4_]^−^, 2.0 μmol), methylcyclohexane (26 g), ethylene and propylene mixed gas (1:1, 20 bar), 80–90 °C. 50 min. ^b^ TOA (trioctylaluminum) or MMAO (modified-methylaluminoxane) was employed as a scavenger. ^c^ (Octyl)_2_Zn was employed as a chain transfer agent. ^d^ Propylene content measured by ^1^H-NMR spectra. ^e^ Measured by GPC at 160 °C, using trichlorobenzene and calculated relative to PS standards. ^f^ PO-equivalent value converted by the equation ‘*M*_PO_ = 0.495 × *M*_PS_^0.990^/(1 – S)’, where S is the mass fraction of the CH_3_–side chains, i.e., S = (15 × [C_3_H_6_])/[(1 – [C_3_H_6_]) × 28 + ([C_3_H_6_] × 42)] and the expected value calculated by ‘yield (g)/(2 × Zn-mol)’ are written in the parenthesis.

## Supplementary Materials

The following are available online at https://www.mdpi.com/2073-4360/11/7/1093/s1, Figures S1–S14: ^1^H and ^13^C NMR spectra of **1**–**6** and **9**–**16**; Figures S15–S26: ^1^H NMR spectra of the activated complexes with [(C_18_H_37_)_2_N(H)Me]^+^[B(C_6_F_5_)_4_]^−^; Figures S27 and S28: ^1^H NMR spectra of the anhydrous and the water-containing [(C_18_H_37_)_2_N(H)Me]^+^[B(C_6_F_5_)_4_]^−^; Figures S29 and S30: ^1^H NMR spectra of polymers; Figure S31: DSC thermograms.
