# Supplementary material for: Preparation of Half- and Post-Metallocene Hafnium Complexes with Tetrahydroquinoline and Tetrahydrophenanthroline Frameworks for Olefin Polymerization
Source: Polymers (Basel). 2019 Jun 27;11(7):1093. doi: 10.3390/polym11071093 (PMC6680767; doi:10.3390/polym11071093)

Supporting Information

Preparation of Half- and Post-metallocene Hafnium Complexes with Tetrahydroquinoline and Tetrahydrophenanthroline Frameworks for Olefin Polymerization

Jun Won Baek, [1] Su Jin Kwon, [1] Hyun Ju Lee, [1] Tae Jin Kim, [1] Ji Yeon Ryu, [b] Junseong Lee, [2] Eun Ji Shin, [3] Ki Soo Lee, [3] and Bun Yeoul Lee [1,*]

[1] Department of Molecular Science and Technology, Ajou University, Suwon 16499, South Korea; btw91@ajou.ac.kr (J.W.B.); ksj9355@ajou.ac.kr (S.J.K.); hjulee4639@ajou.ac.kr (H.J.L.); playing3457@ajou.ac.kr (T.J.K.)

[2] Department of Chemistry, Chonnam National University, 77 Yongbong-ro, Buk-gu, Gwangju 500-757, South Korea; jy5330@naver.com (J.Y.R.); leespy@chonnam.ac.kr (J.L.)

[3] LG Chem, Ltd, 188, Munji-ro, Yuseong-gu Daejeon 305-738, South Korea; eunjis@lgchem.com (E.J.S.); leekisoo@lgchem.com (K.S.L.)

* Correspondence: bunyeoul@ajou.ac.kr; Tel: 82-31-219-1844

Figure S1. ^{1}H and ^{13}C NMR spectra of **1**.

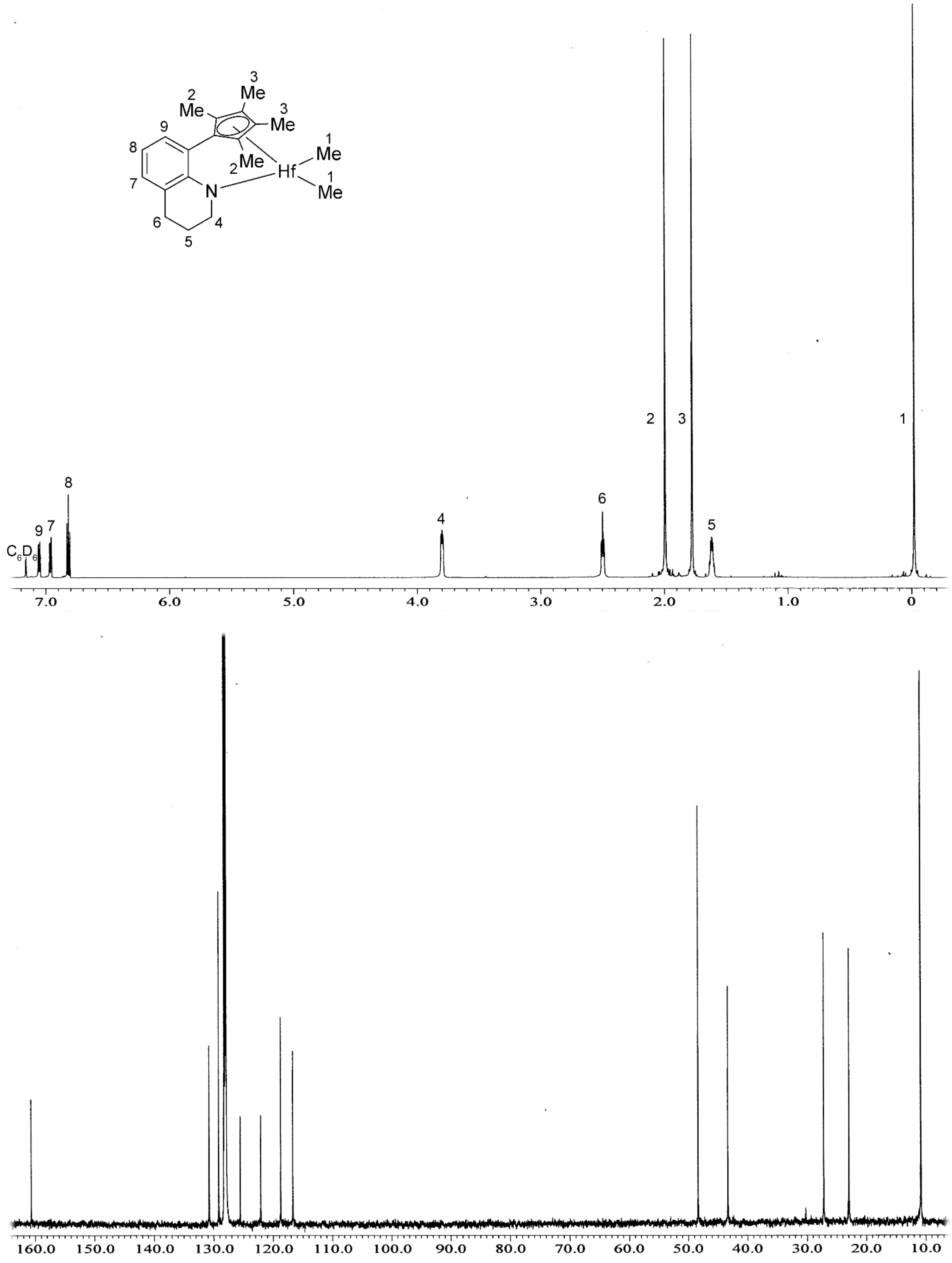

Figure S2. ^{1}H and ^{13}C NMR spectra of **2**.

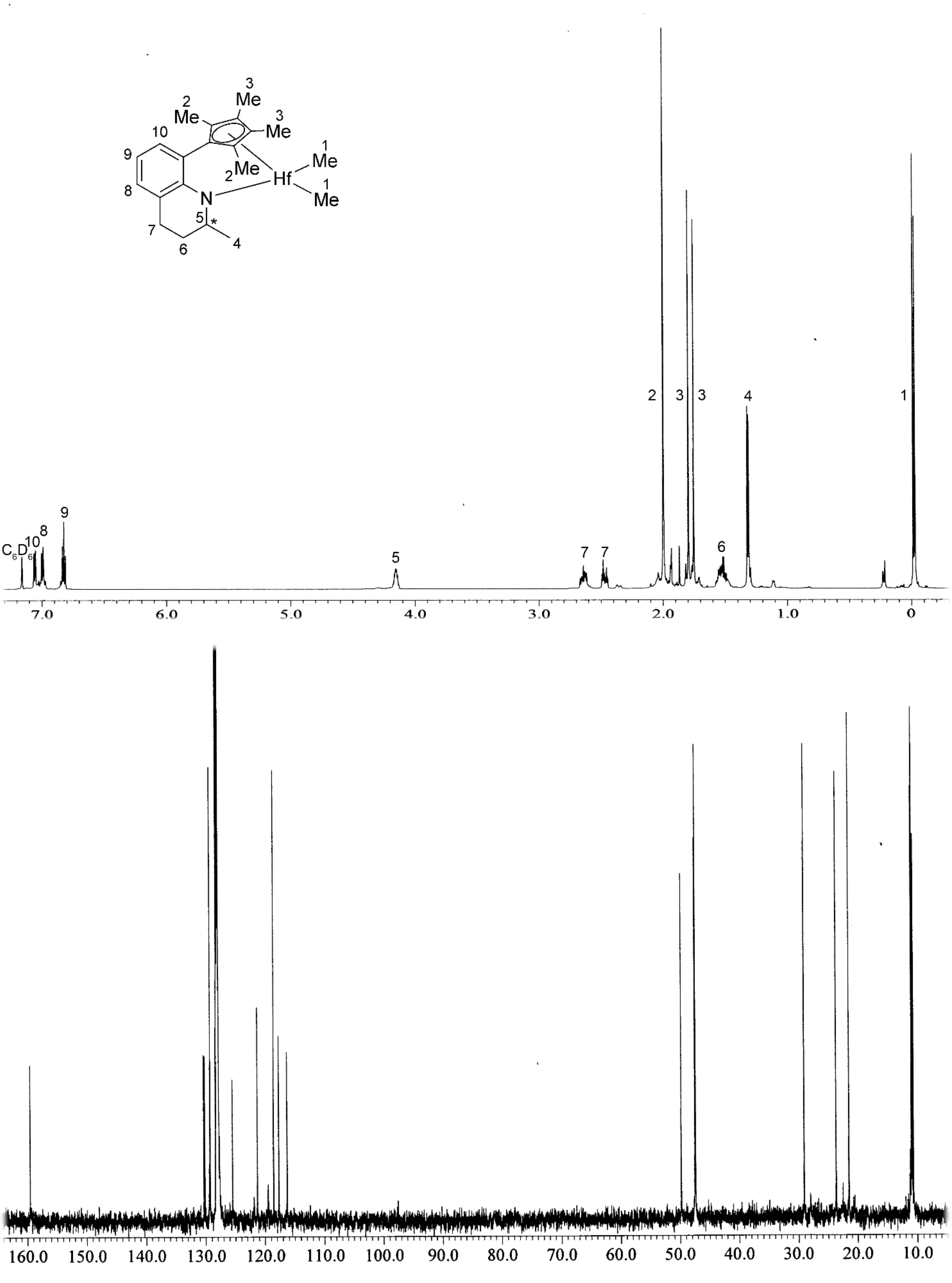

Figure S3. ^{1}H and ^{13}C NMR spectra of **3**.

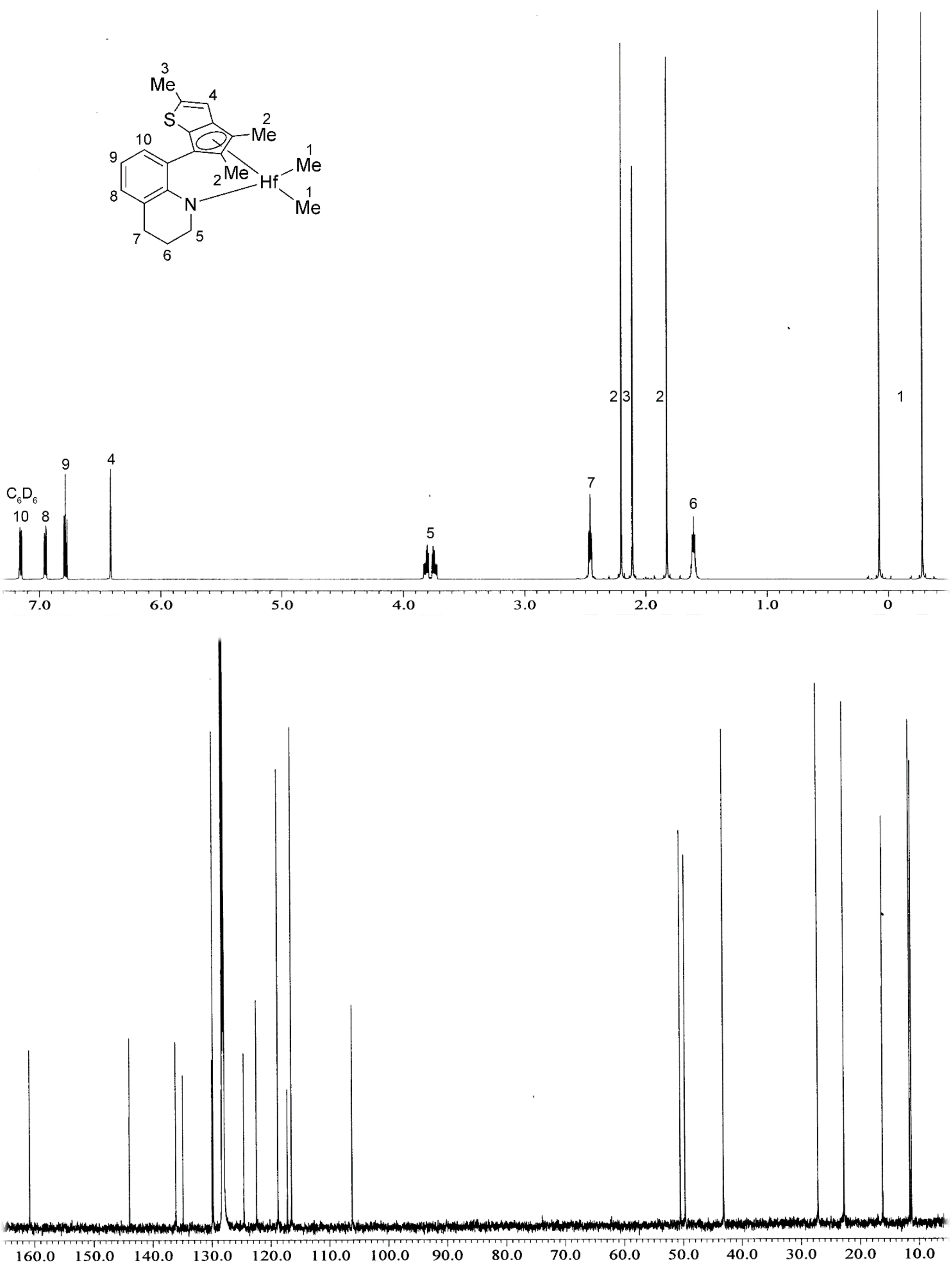

Figure S4. ^{1}H and ^{13}C NMR spectra of **4**.

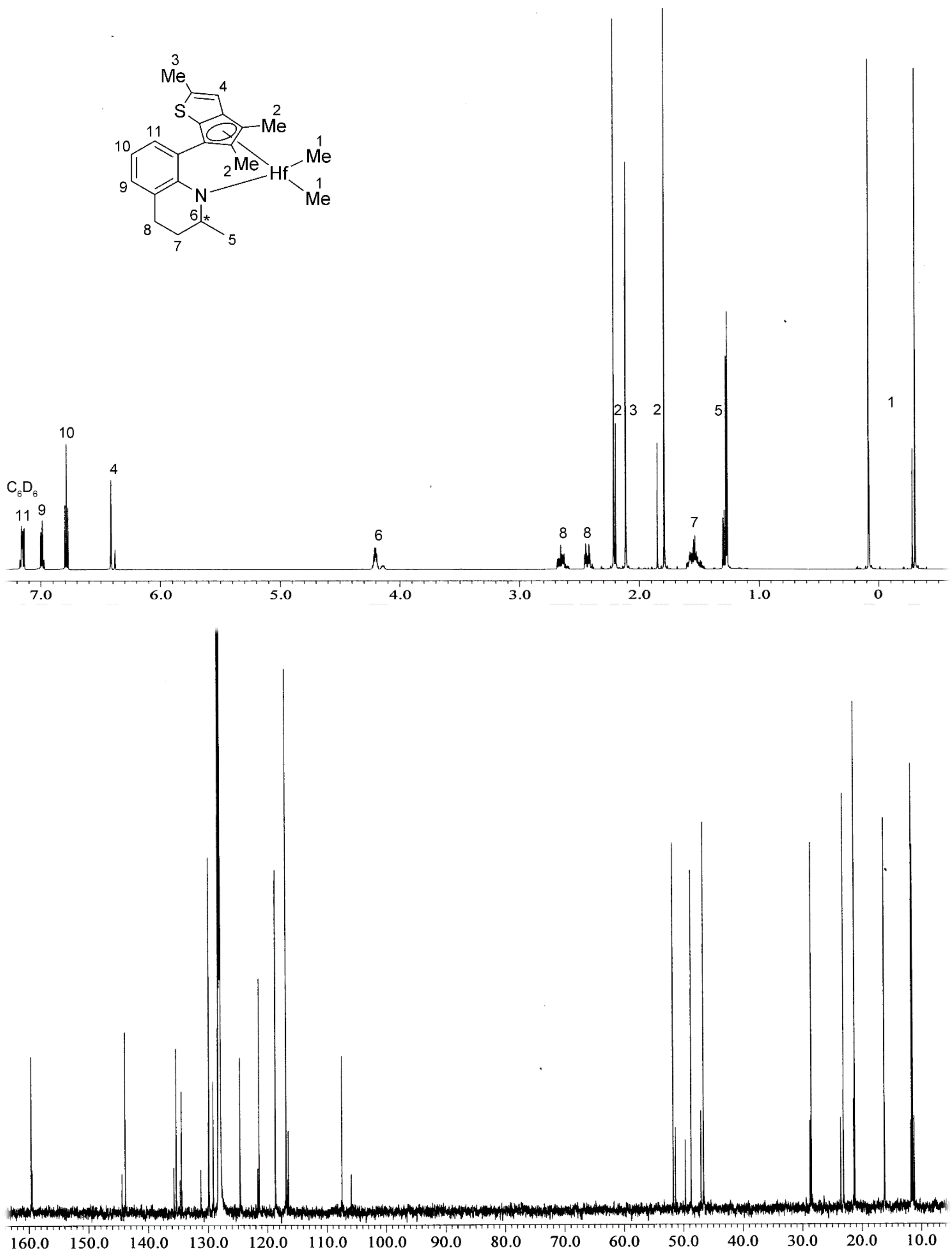

Figure S5. ^{1}H and ^{13}C NMR spectra of **5**.

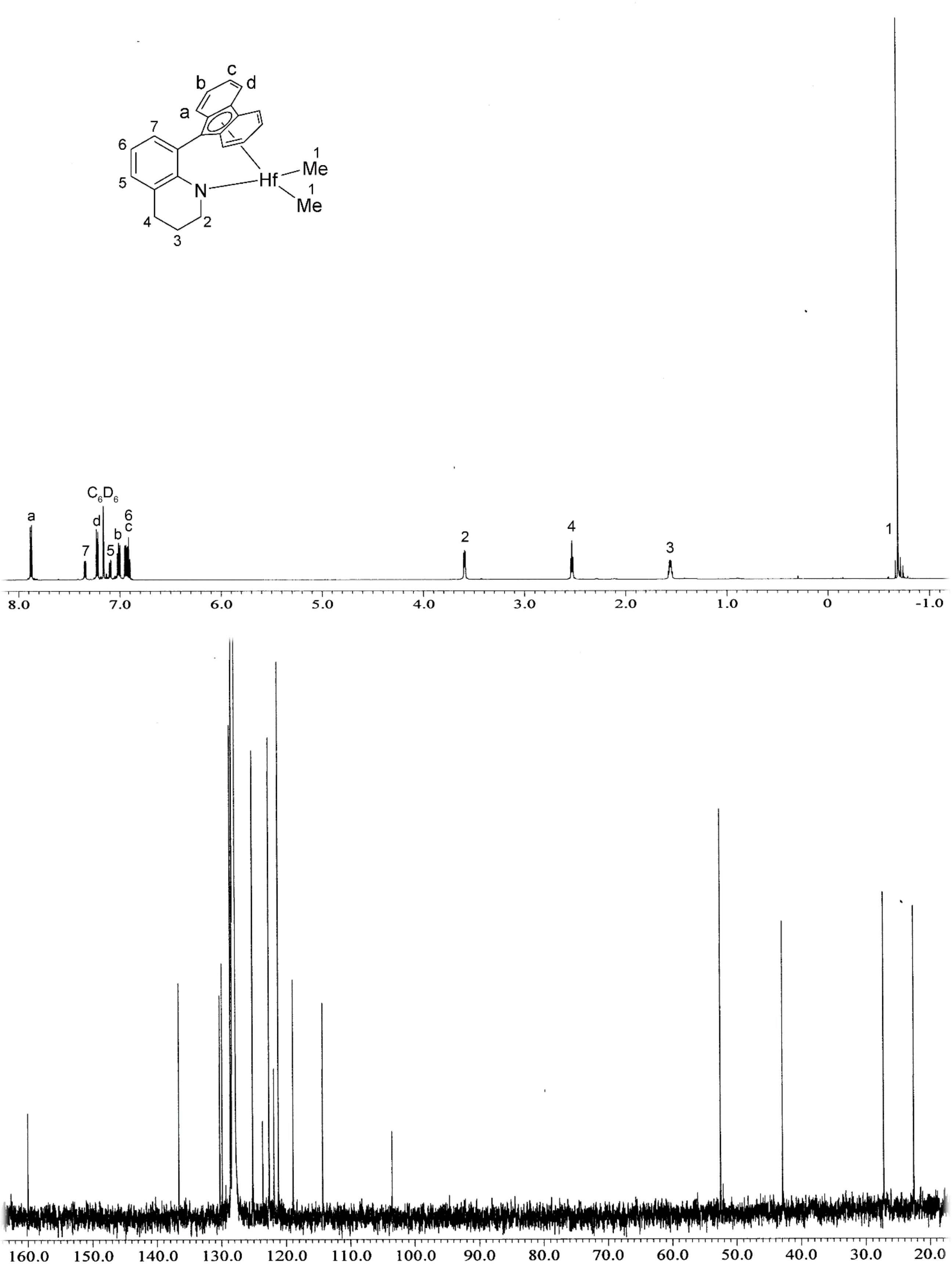

Figure S6. ^{1}H and ^{13}C NMR spectra of **6**.

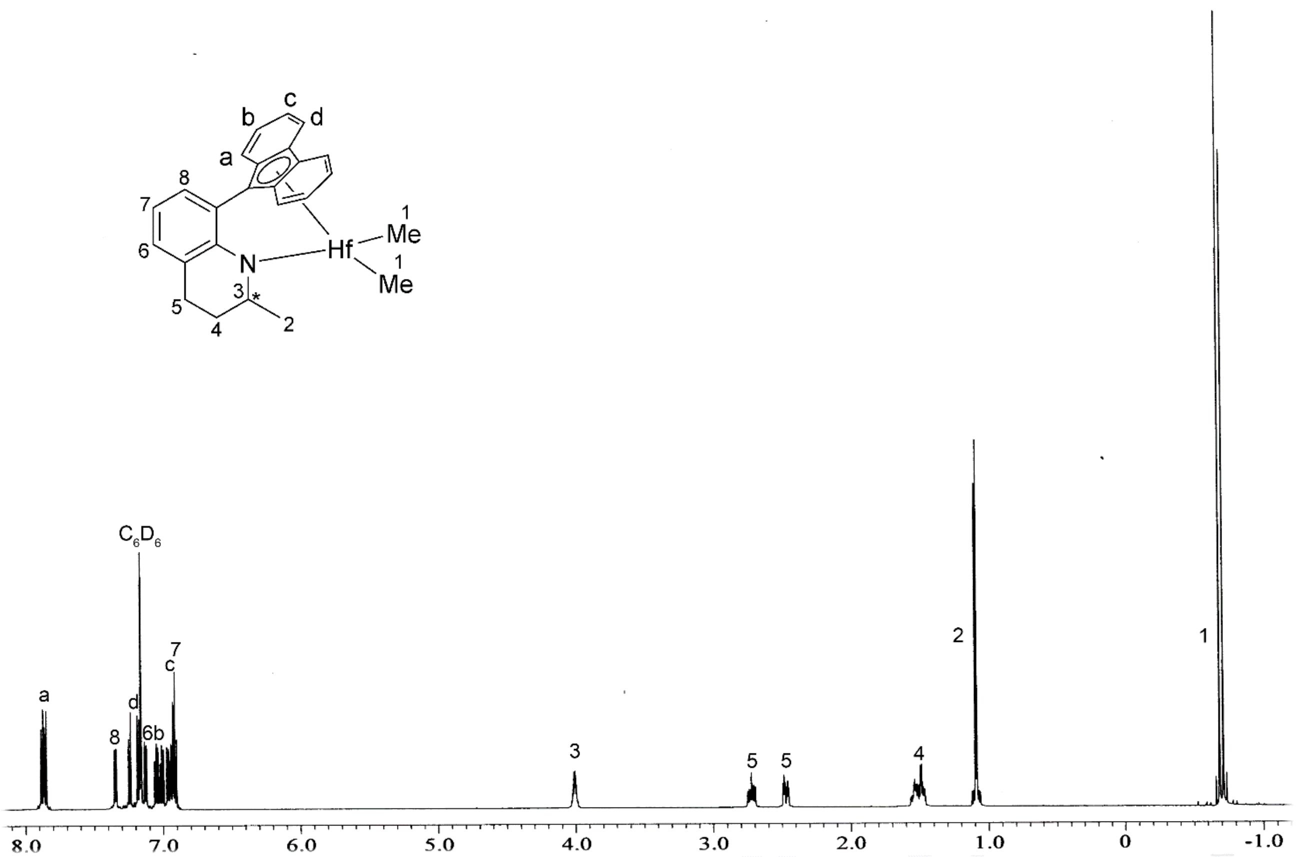

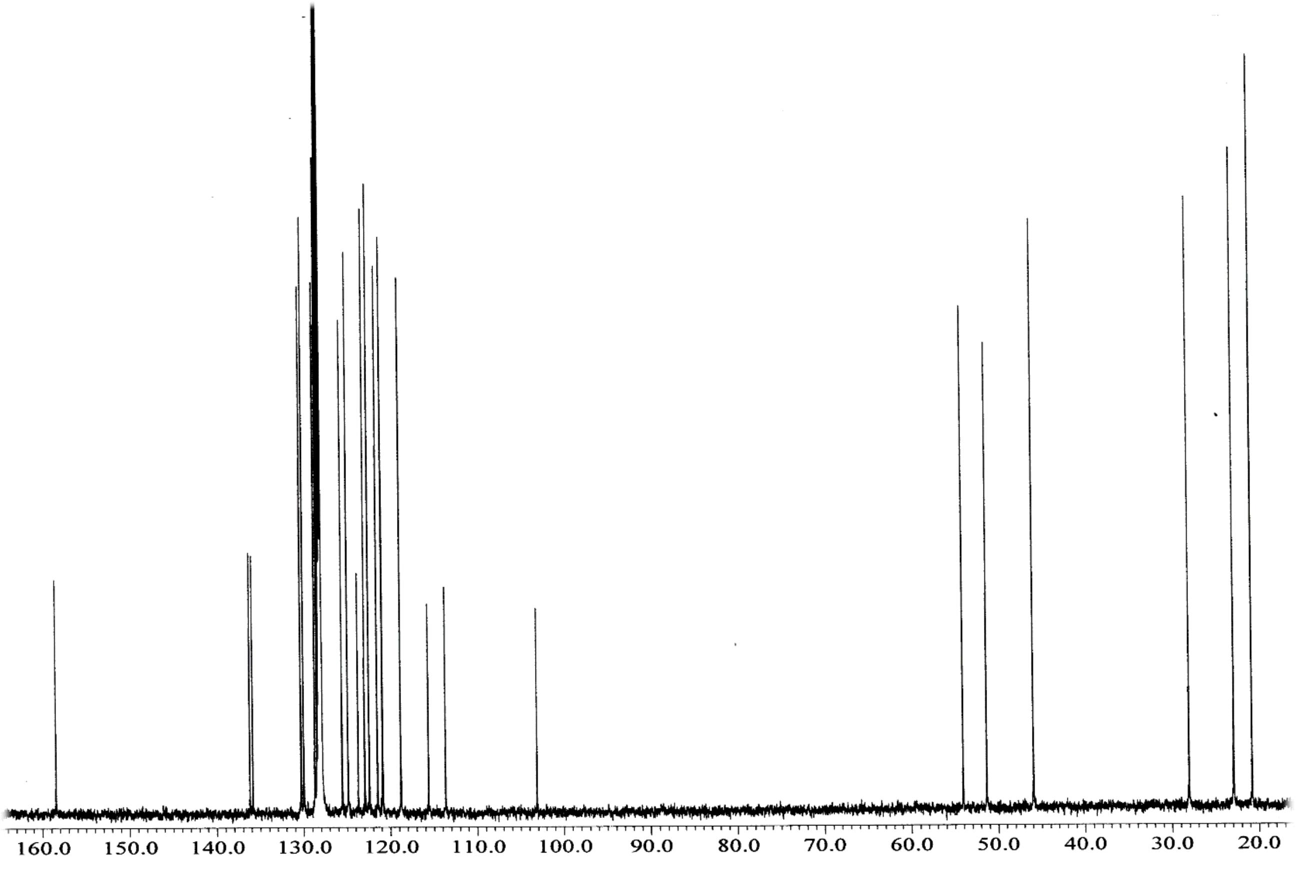

Figure S7. ^{1}H and ^{13}C NMR spectra of **9**.

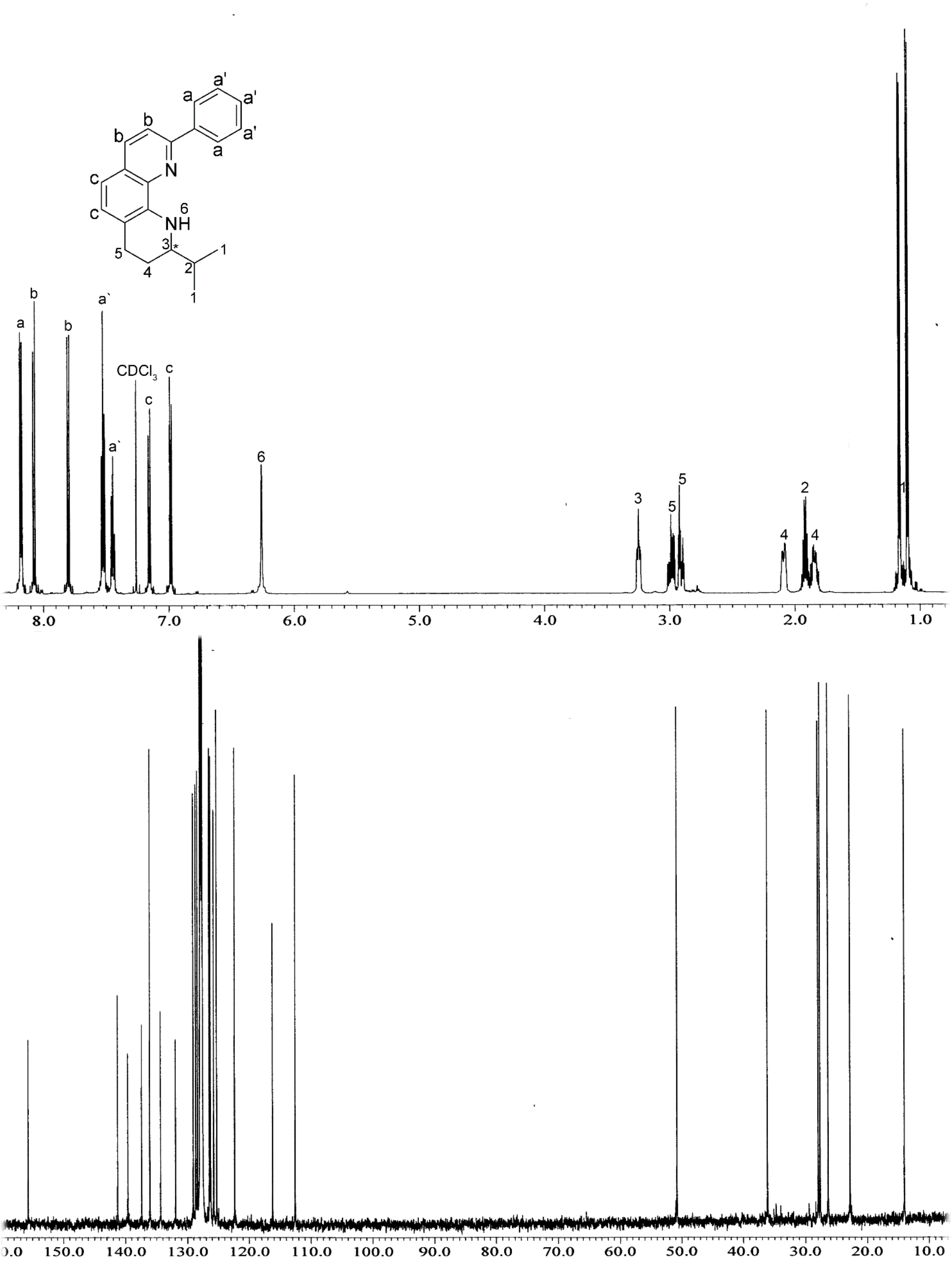

Figure S8. ^{1}H and ^{13}C NMR spectra of **10**.

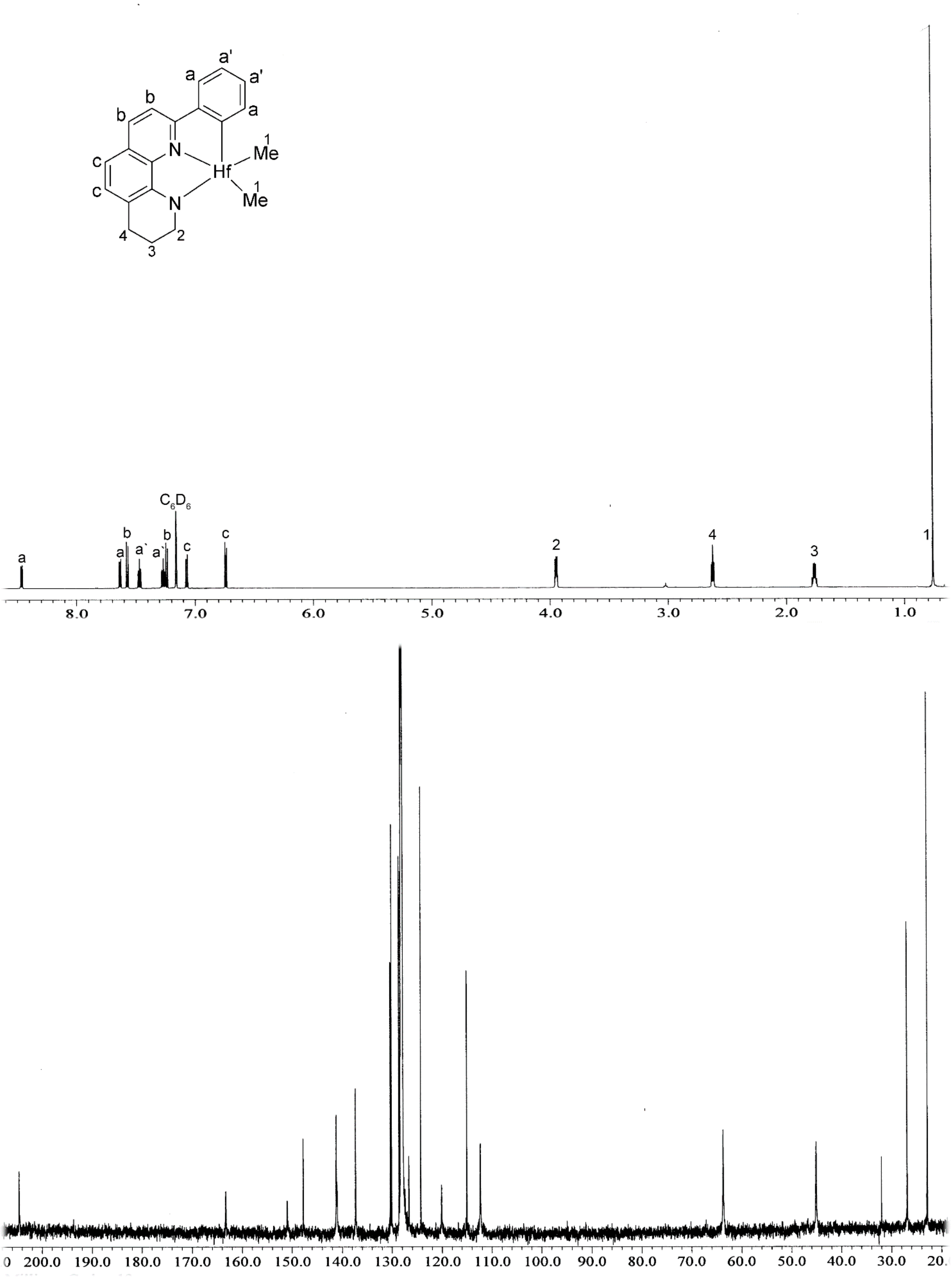

Figure S9. ^{1}H and ^{13}C NMR spectra of **11**.

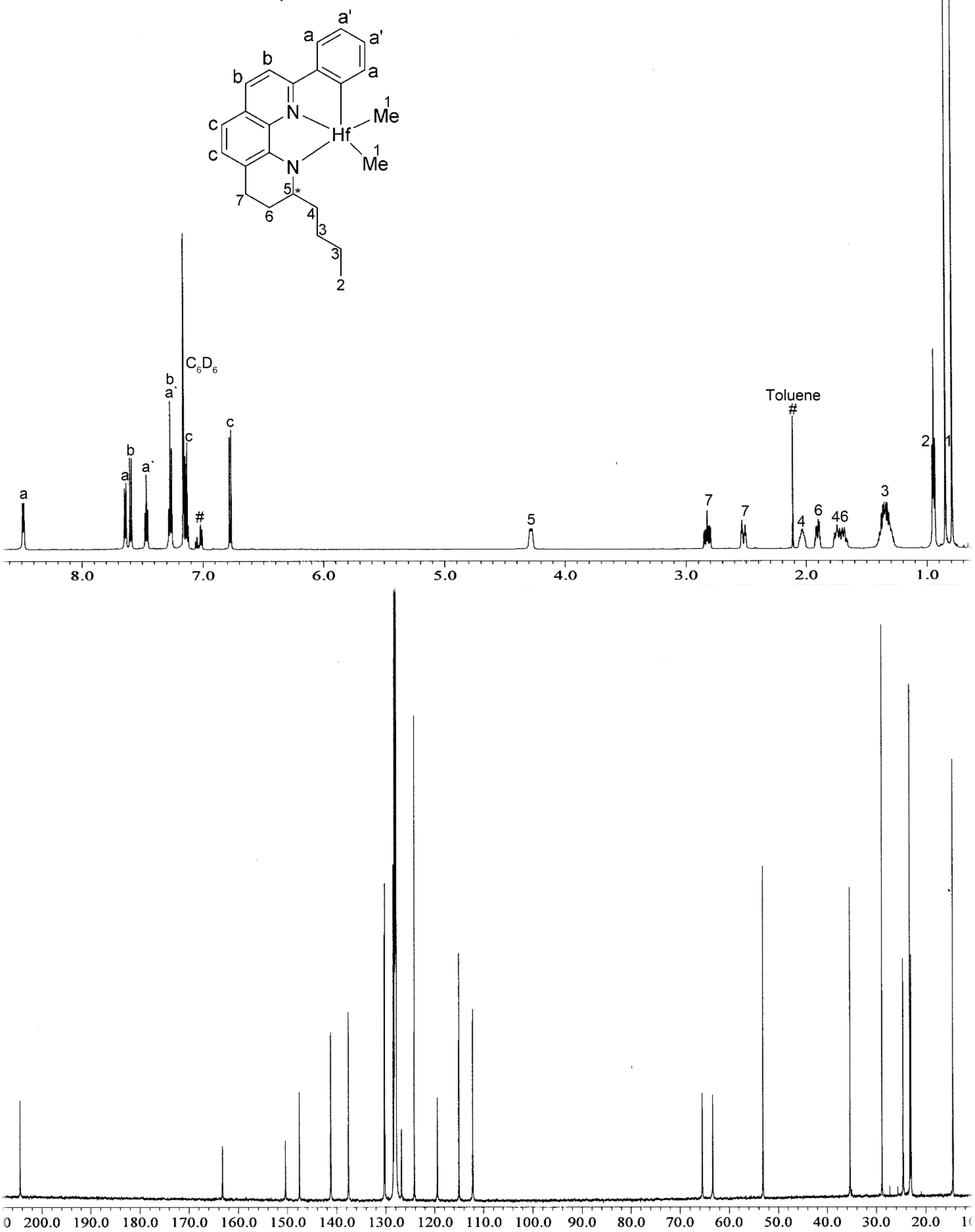

Figure S10. ^{1}H and ^{13}C NMR spectra of **12**.

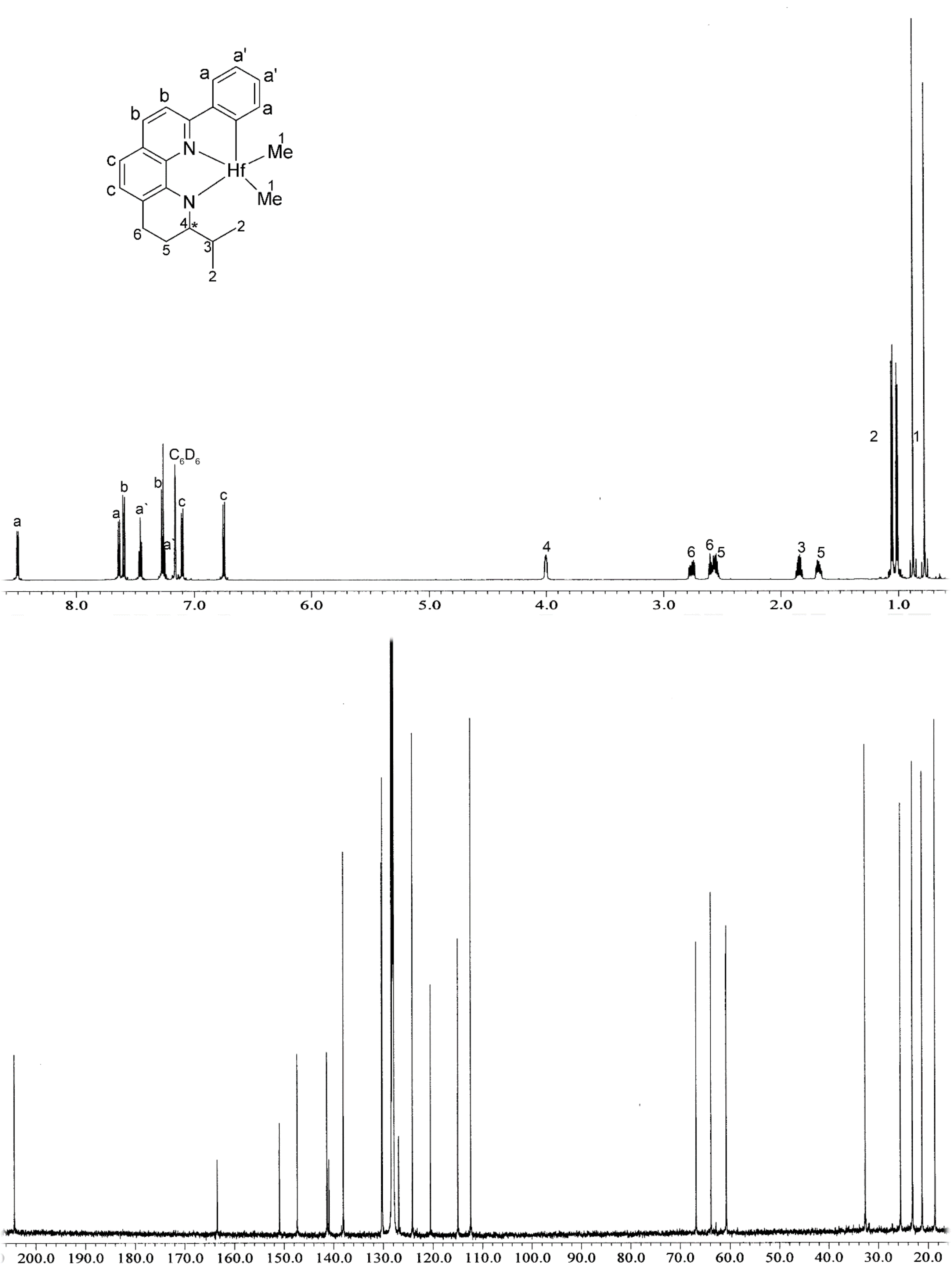

Figure S11. ^{1}H and ^{13}C NMR spectra of **13**.

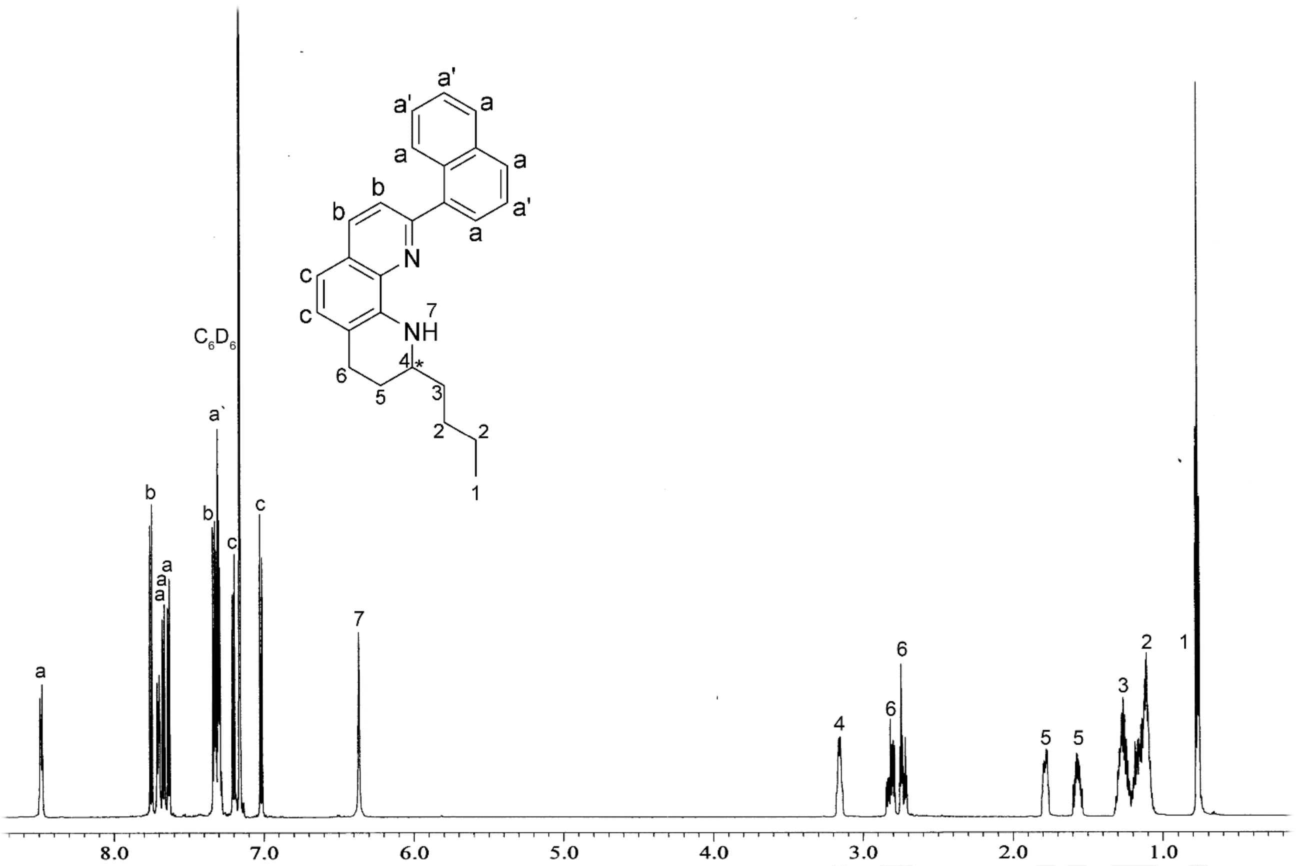

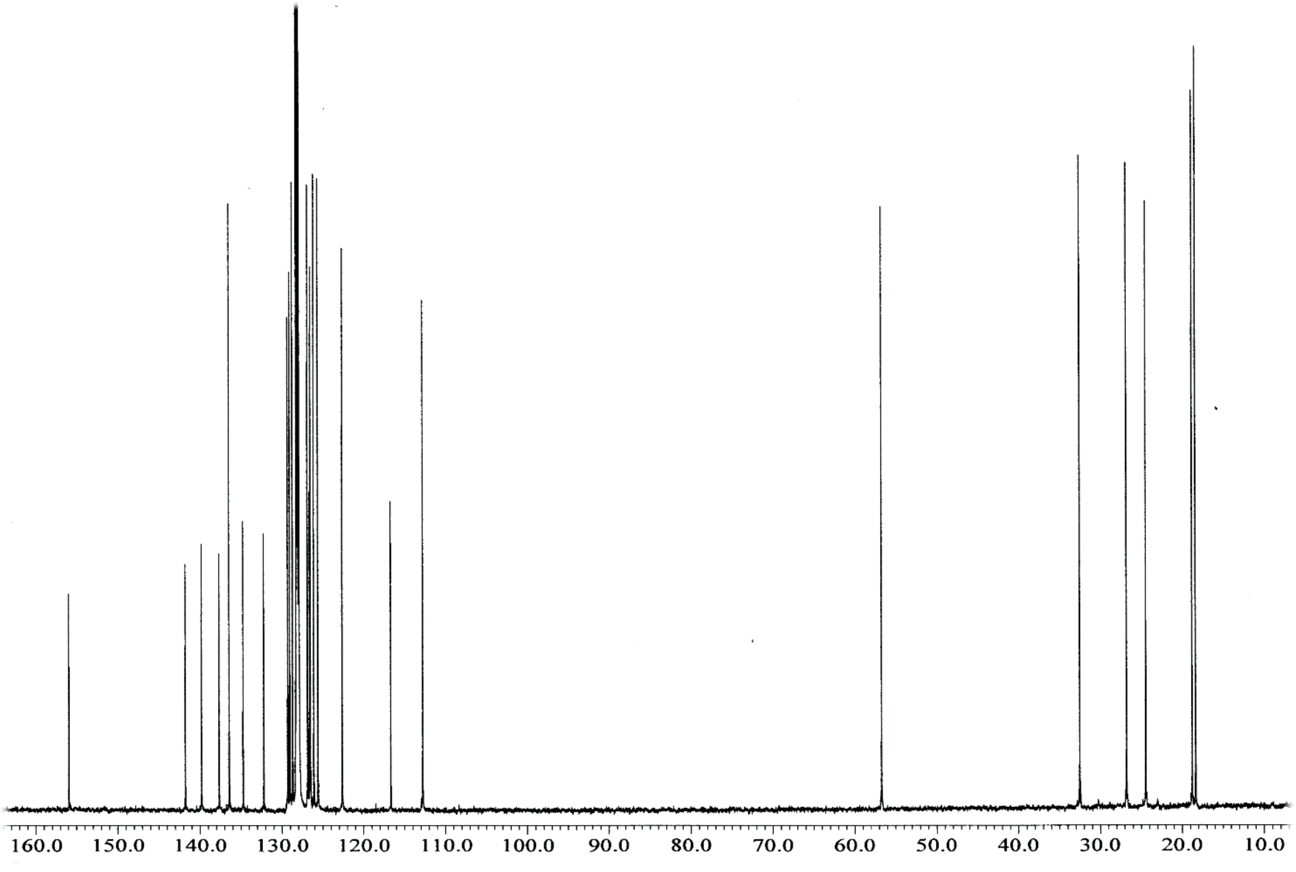

Figure S12. ^{1}H and ^{13}C NMR spectra of **14**.

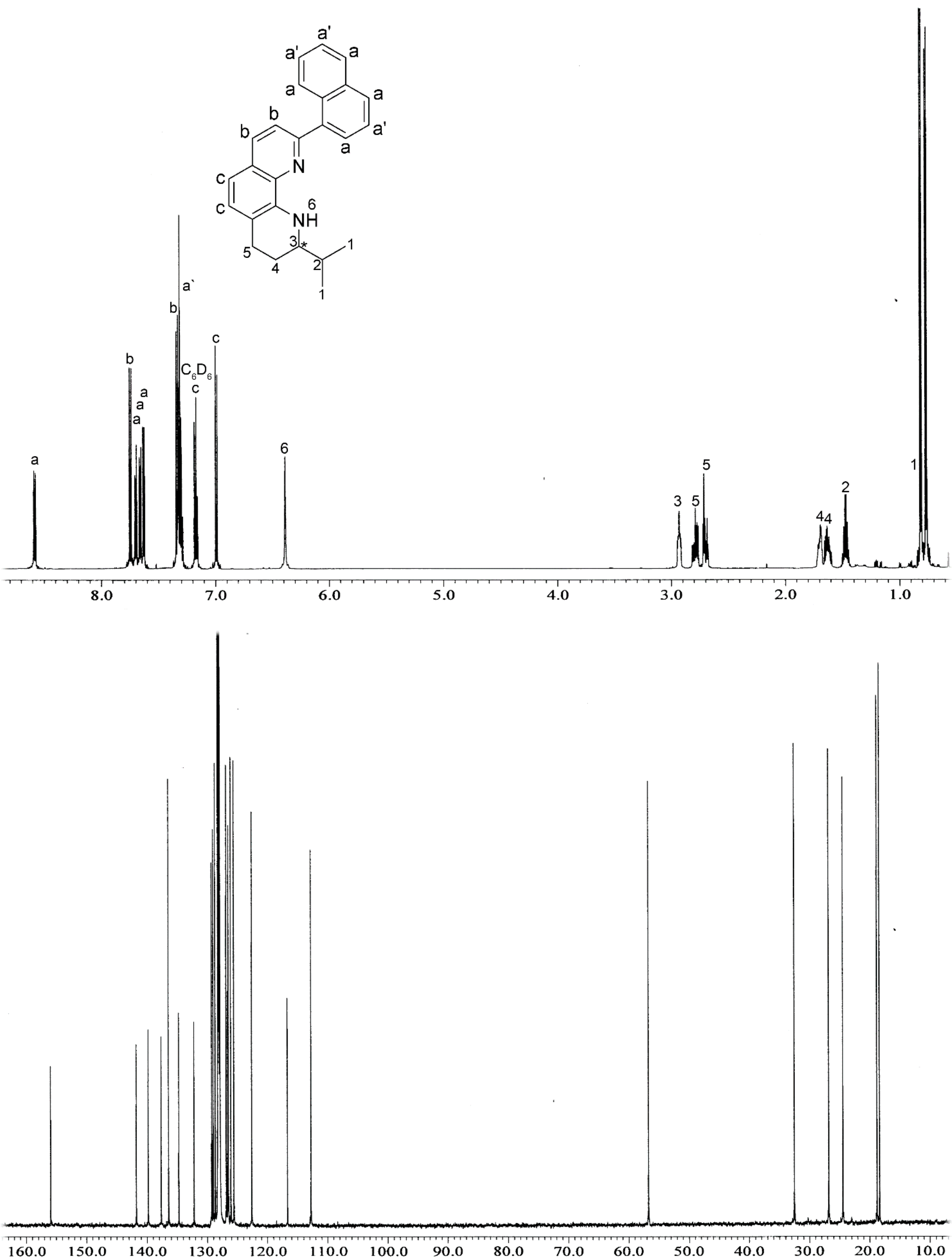

Figure S13. ^{1}H and ^{13}C NMR spectra of **15**.

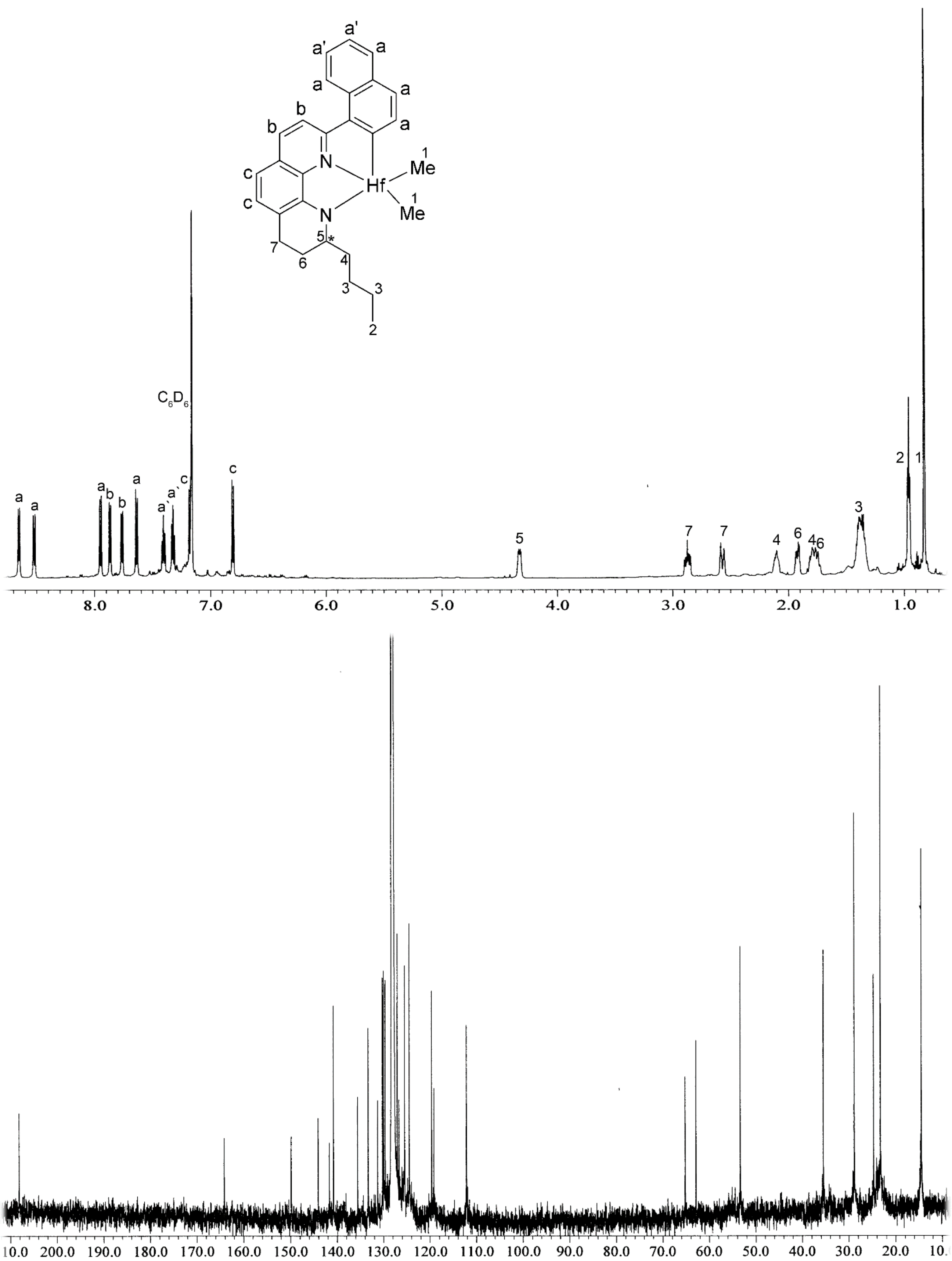

Figure S14. ^{1}H and ^{13}C NMR spectra of **16**.

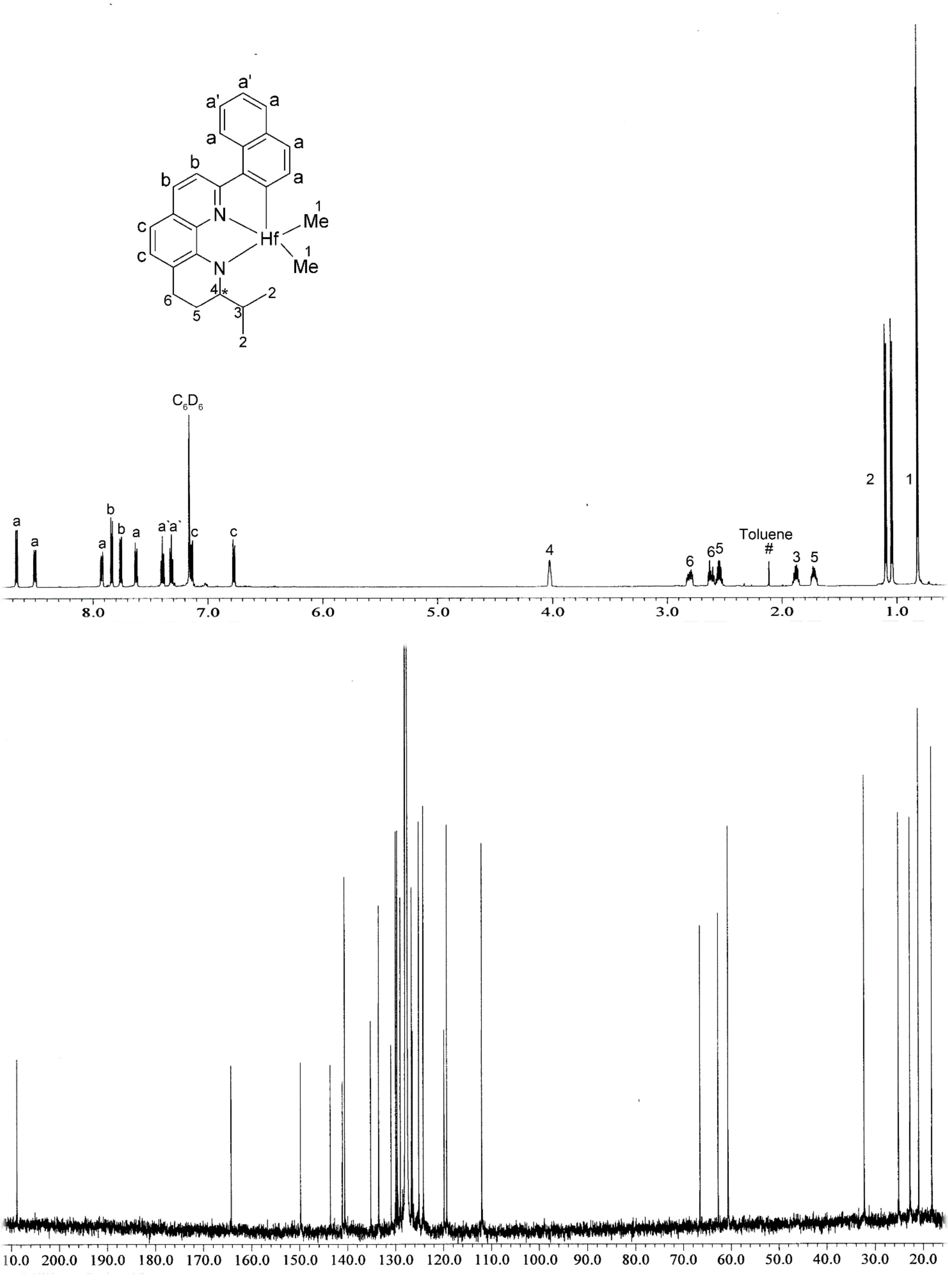

Figure S15. ^{1}H and ^{13}C NMR spectra recorded on the reaction of **1** with $[(C_{18}H_{37})_2N(H)Me]^+[B(C_6F_5)_4]^-$ at 3 h.

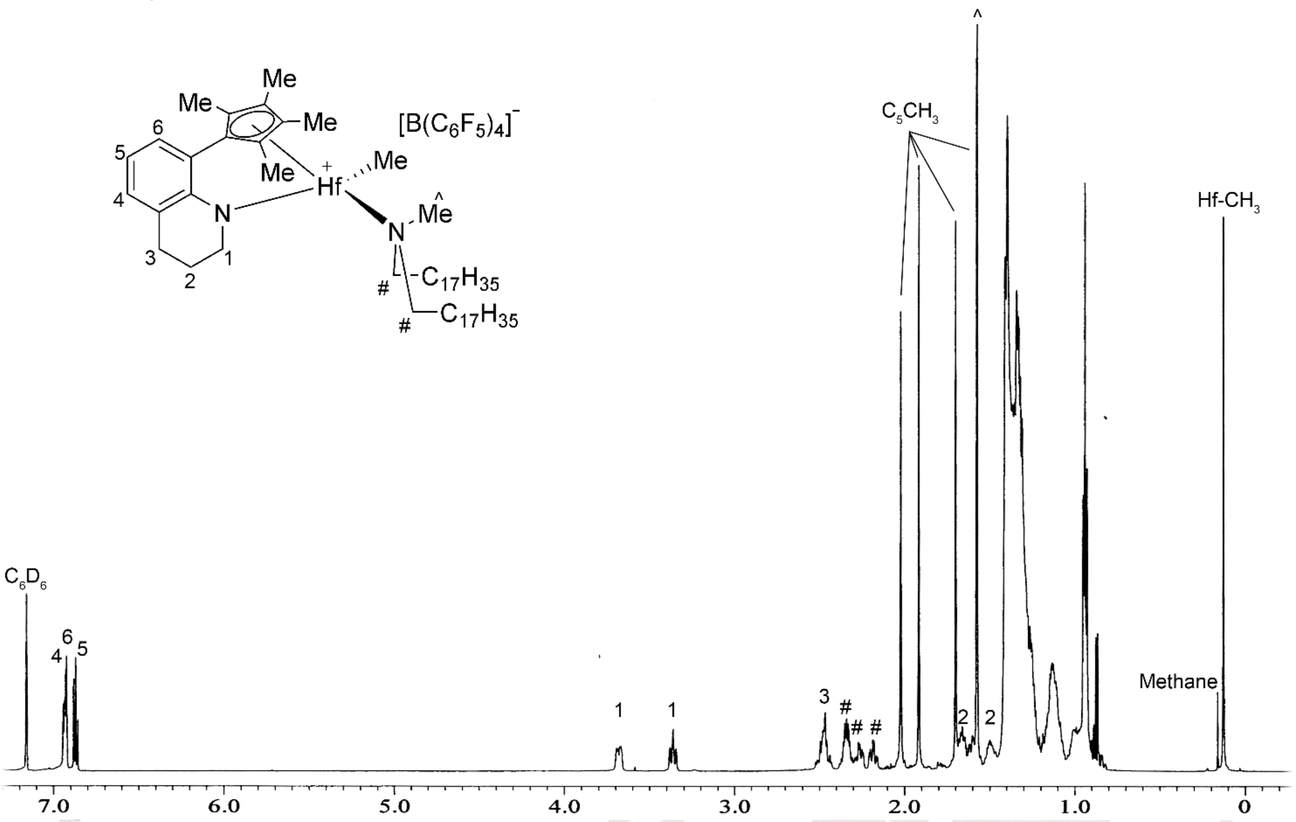

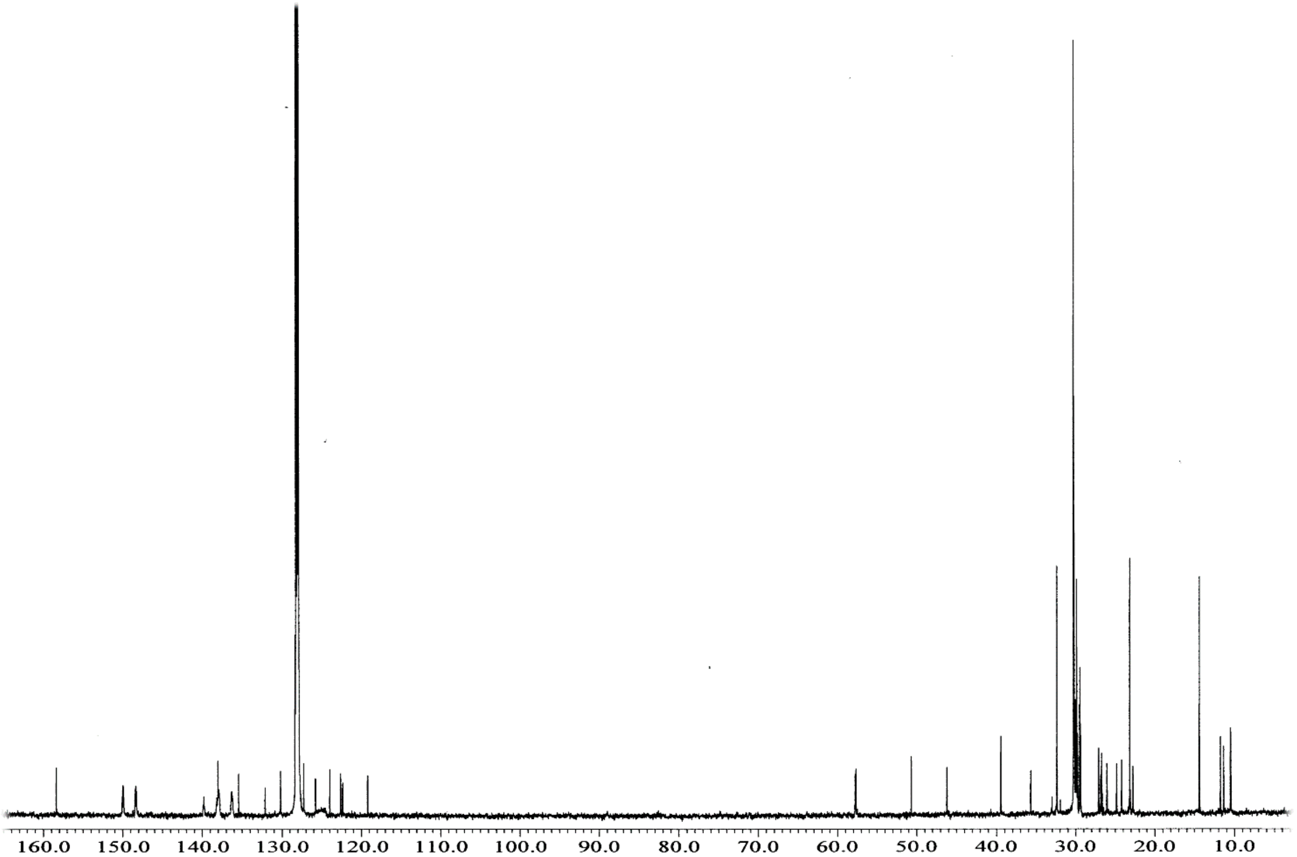

Figure S16. ^{1}H NMR spectrum recorded on the reaction of **2** with $[(C_{18}H_{37})_2N(H)Me]^{+}[B(C_6F_5)_4]^{-}$ at 3 h.

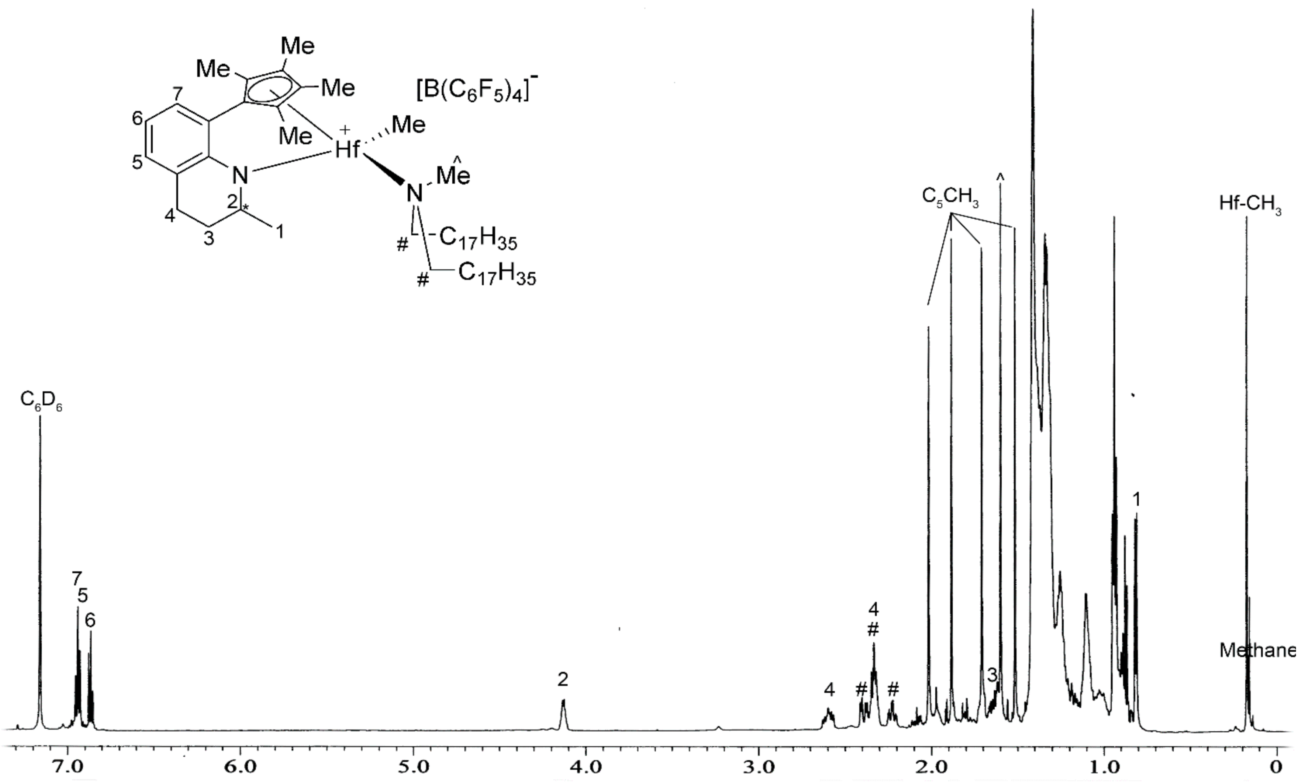

Figure S17. ^{1}H NMR spectrum recorded on the reaction of **3** with $[(C_{18}H_{37})_2N(H)Me]^{+}[B(C_6F_5)_4]^{-}$ at 5 h.

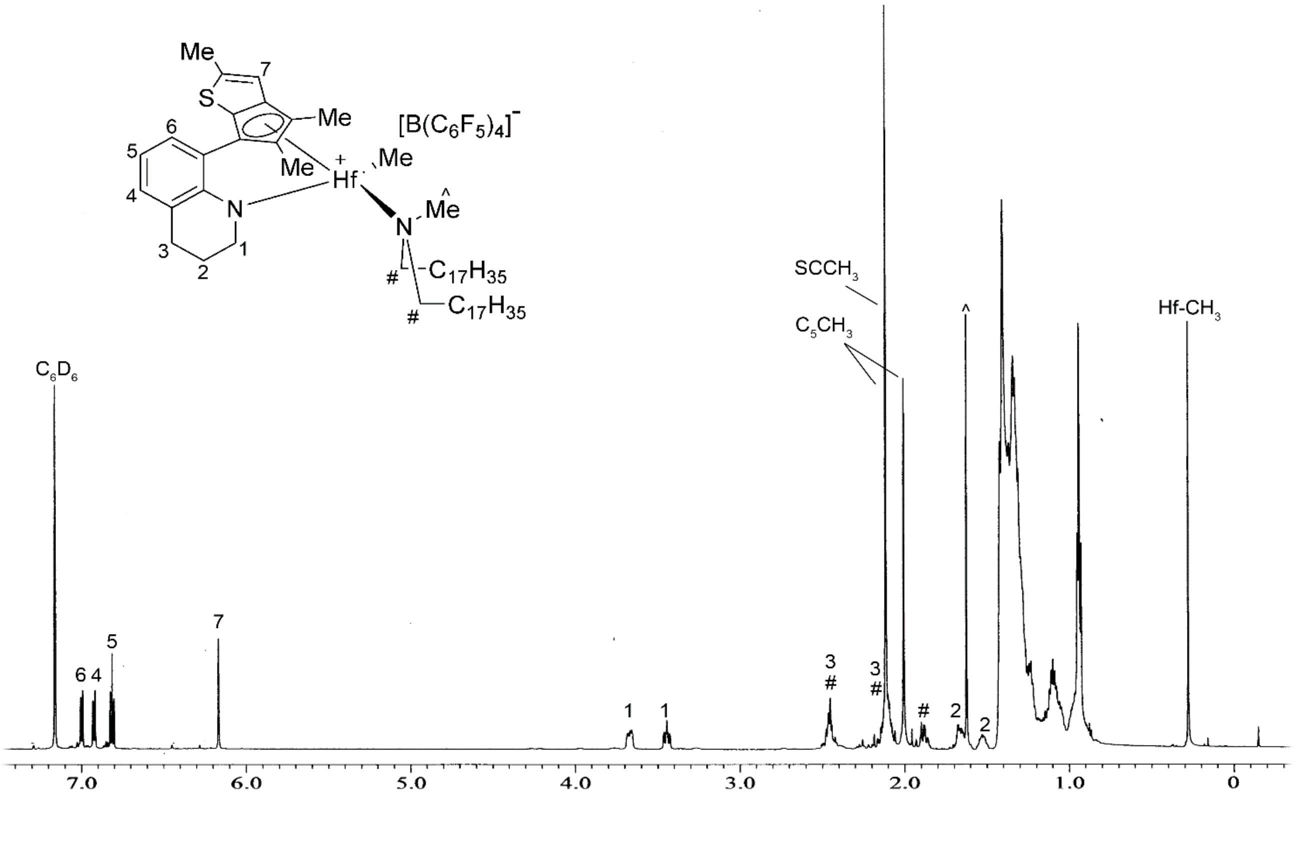

Figure S18. ^{1}H NMR spectrum recorded on the reaction of **4** with $[(C_{18}H_{37})_2N(H)Me]^+[B(C_6F_5)_4]^-$ at 5 h.

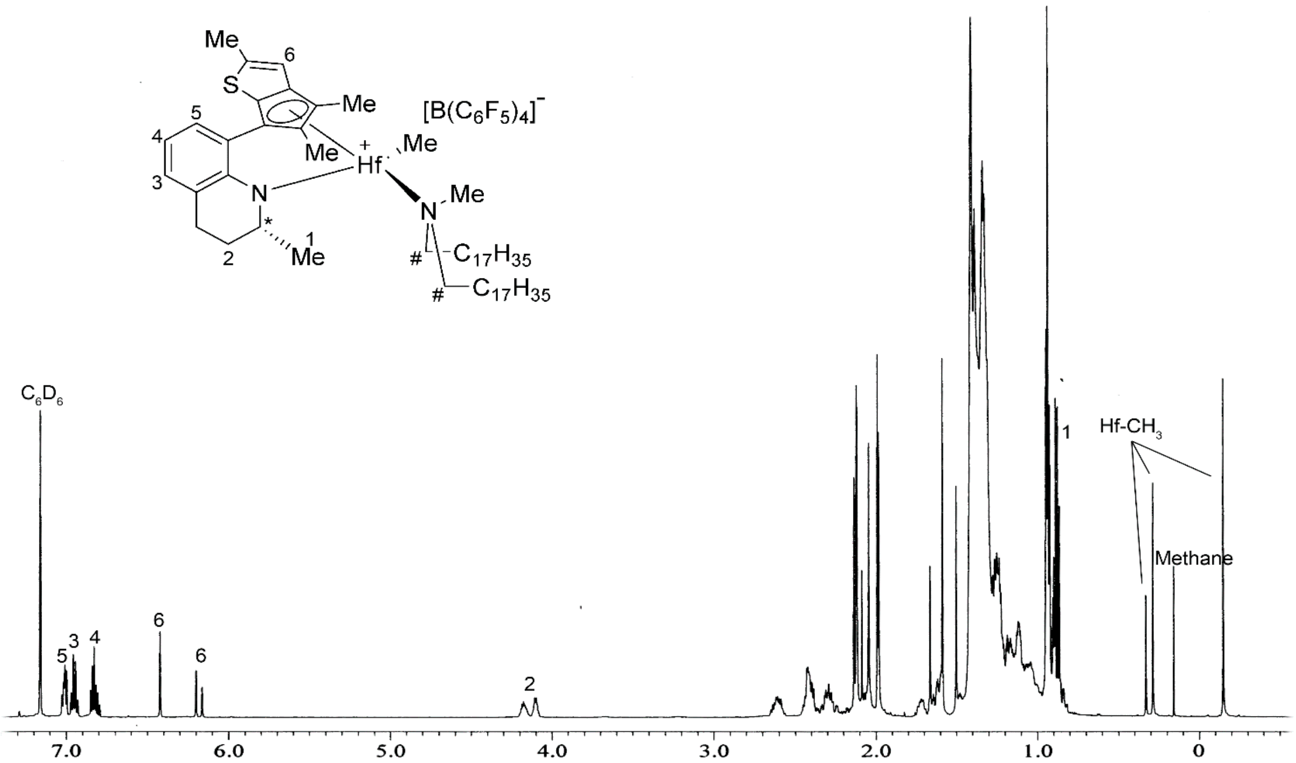

Figure S19. ^{1}H NMR spectrum recorded on the reaction of **5** with $[(C_{18}H_{37})_2N(H)Me]^+[B(C_6F_5)_4]^-$ at 3 h.

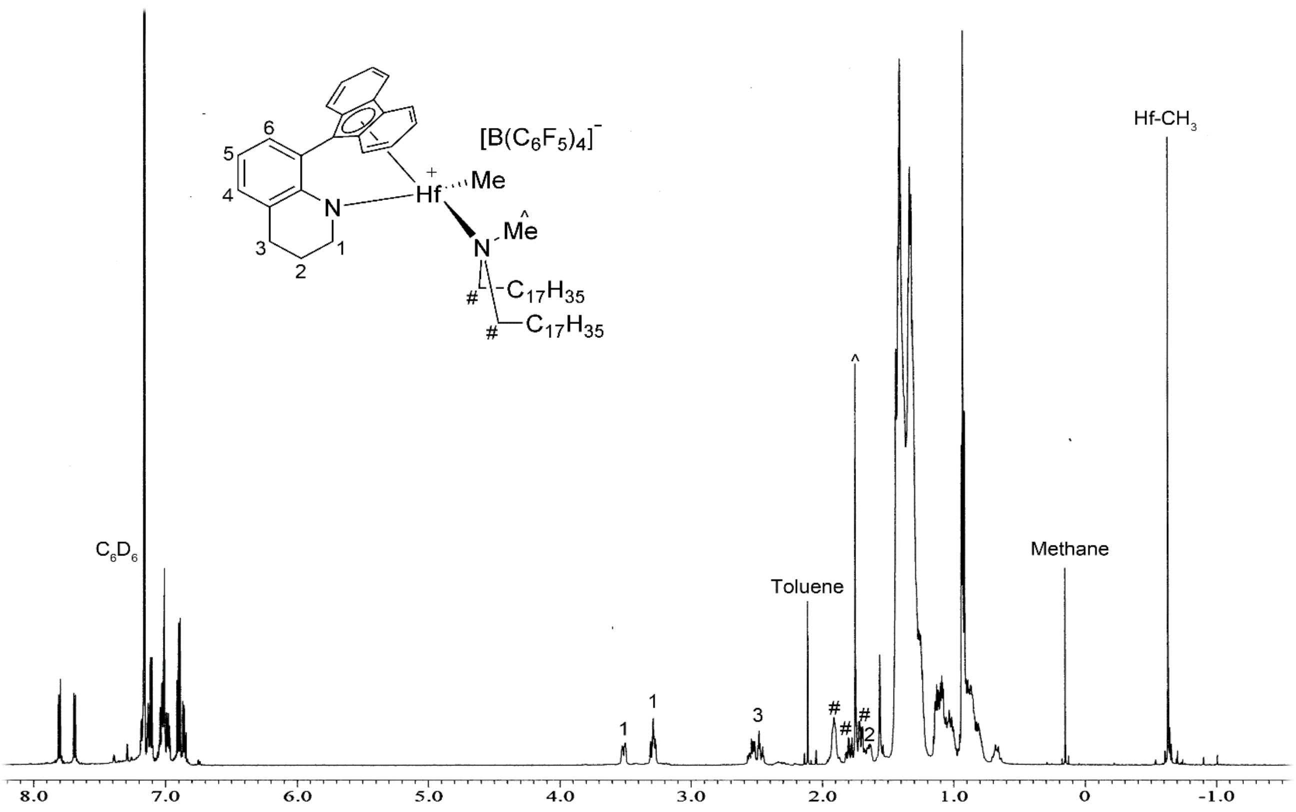

Figure S20. ^{1}H NMR spectrum recorded on the reaction of **6** with $[(C_{18}H_{37})_2N(H)Me]^+[B(C_6F_5)_4]^-$ at 3 h.

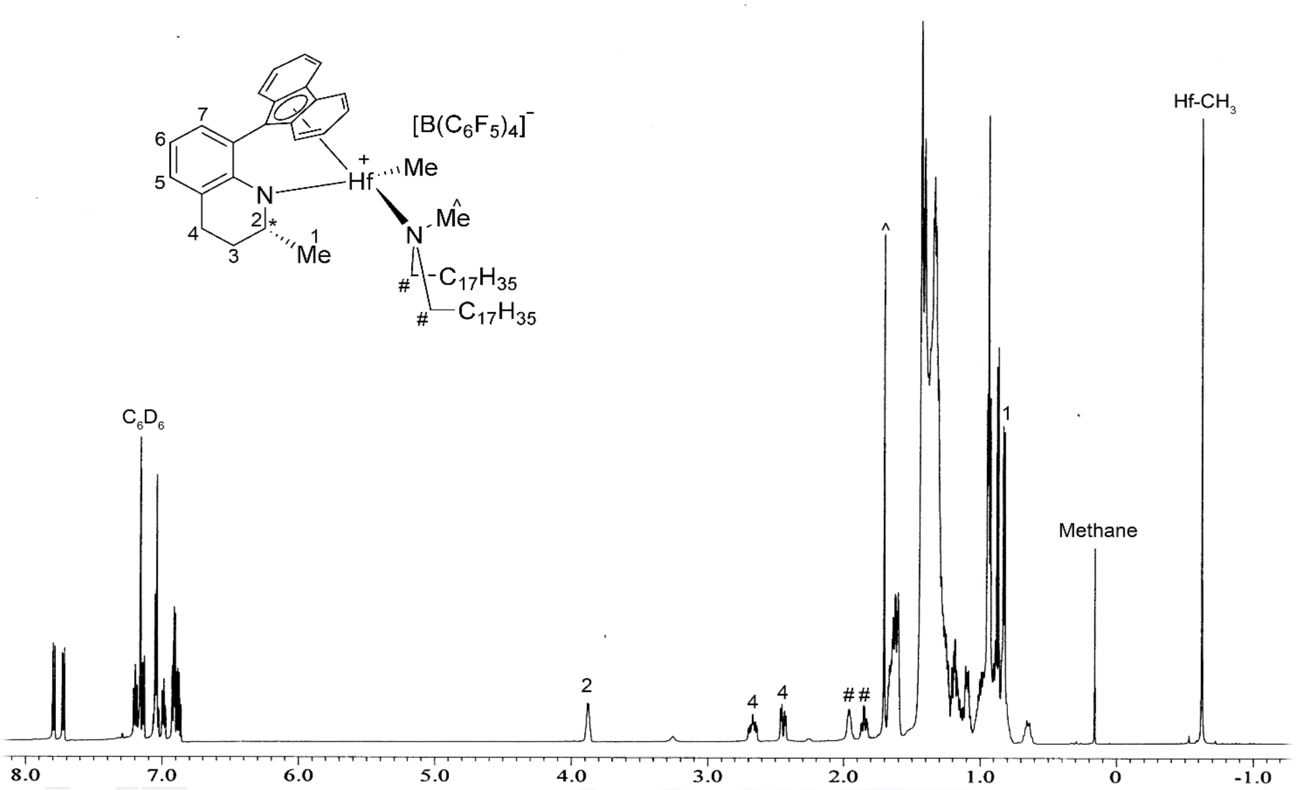

Figure S21. ^{1}H NMR spectrum recorded on the reaction of **10** with $[(C_{18}H_{37})_2N(H)Me]^+[B(C_6F_5)_4]^-$ at 0.5 h.

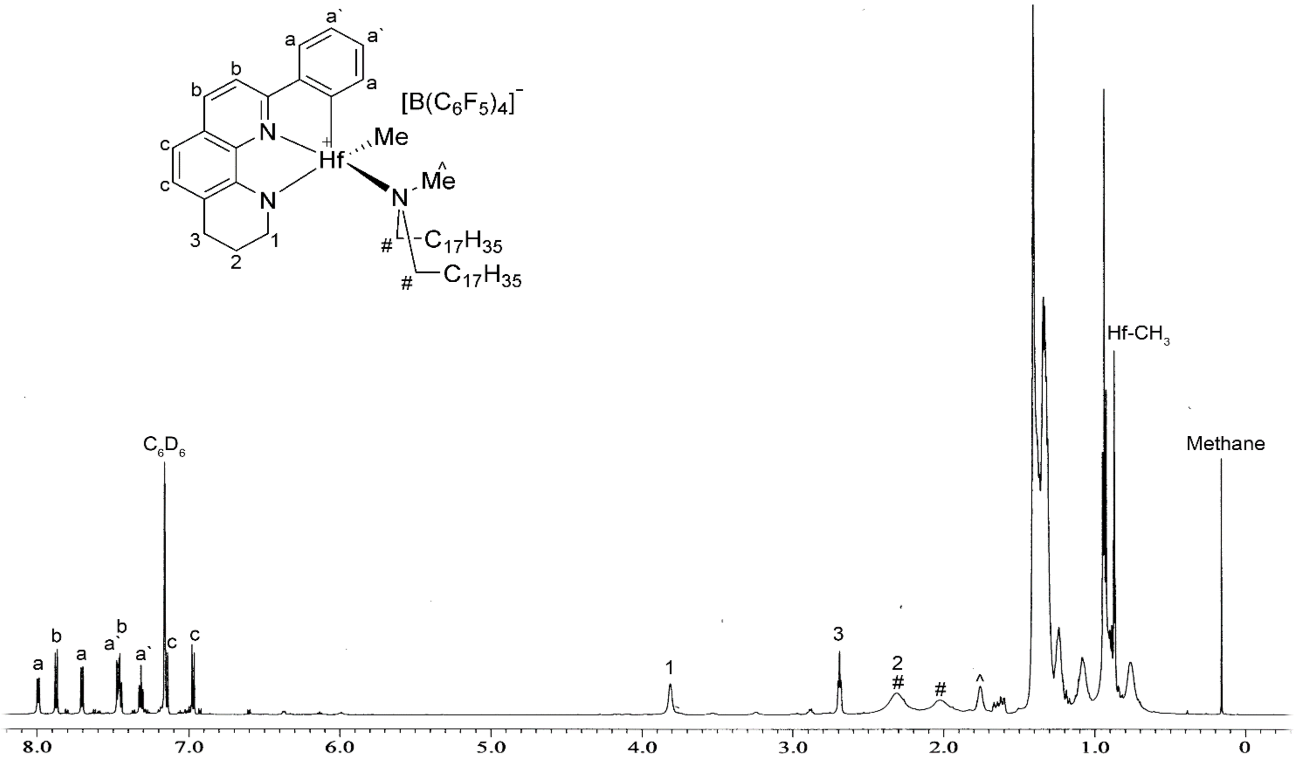

Figure S22. ^{1}H NMR spectrum recorded on the reaction of **11** with $[(C_{18}H_{37})_2N(H)Me]^+[B(C_6F_5)_4]^-$ at 0.5 h.

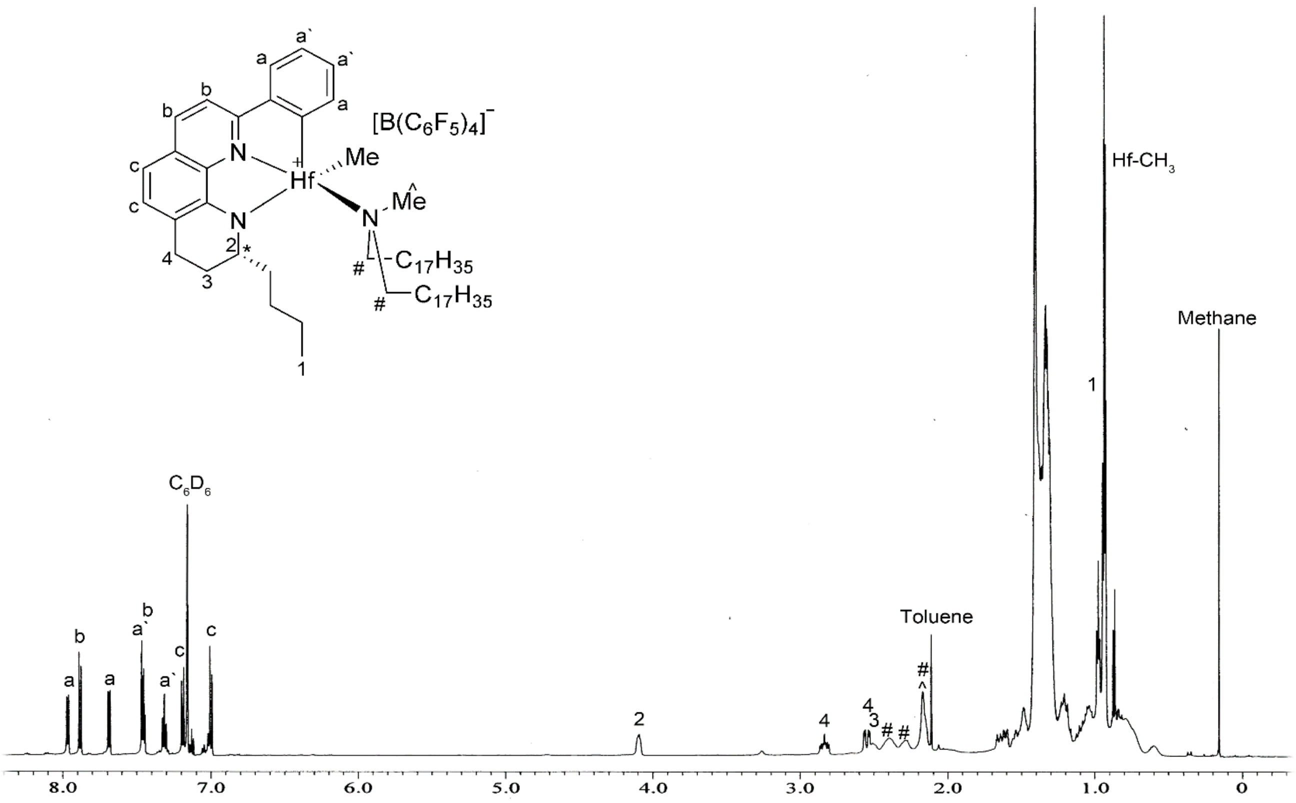

Figure S23. ^{1}H and ^{13}C NMR spectra recorded on the reaction of **12** with $[(C_{18}H_{37})_2N(H)Me]^+[B(C_6F_5)_4]^-$ at 0.5 h.

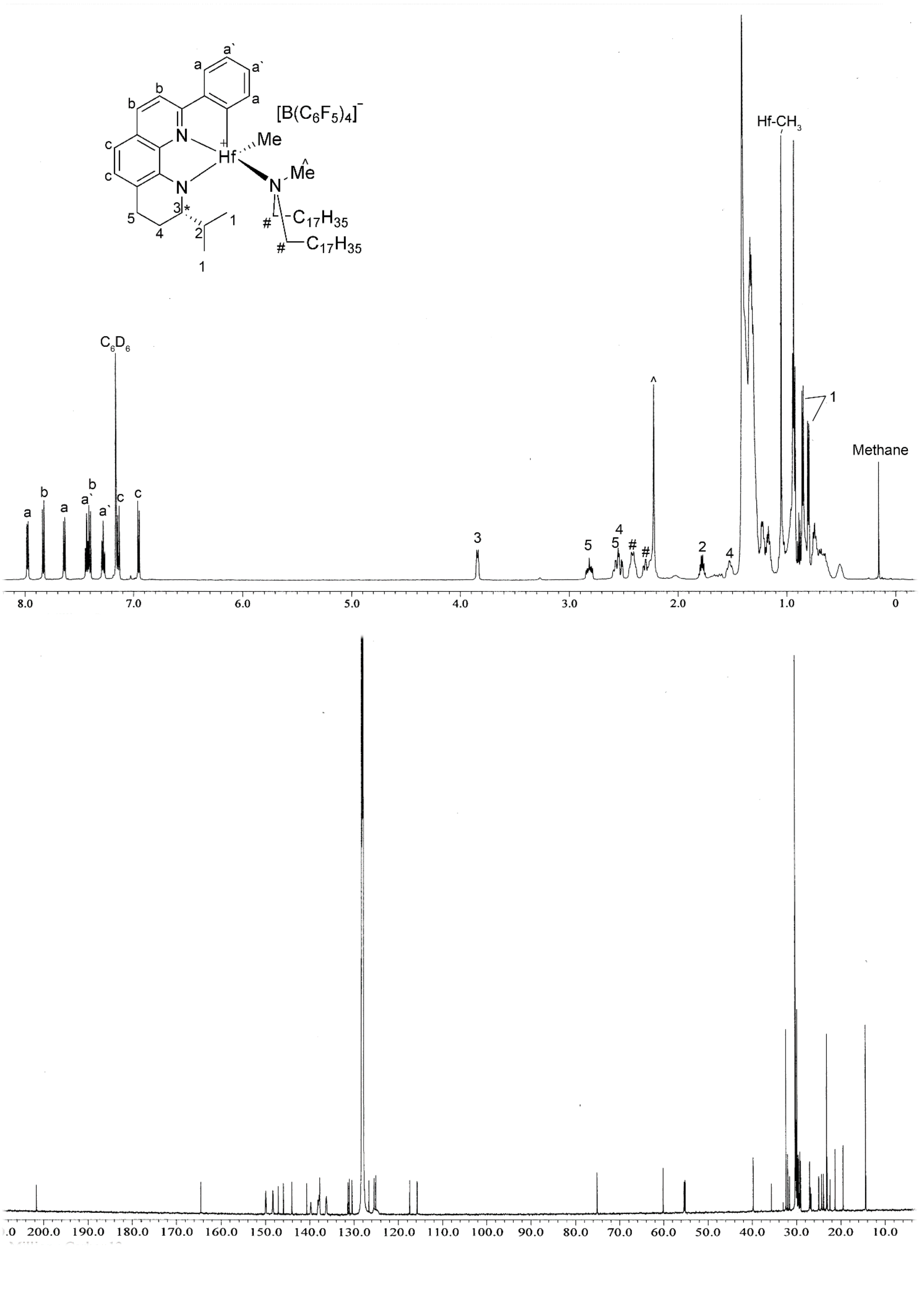

Figure S24. ^{1}H NMR spectrum recorded on the reaction of **15** with $[(C_{18}H_{37})_2N(H)Me]^{+}[B(C_6F_5)_4]^{-}$ at 1 h..

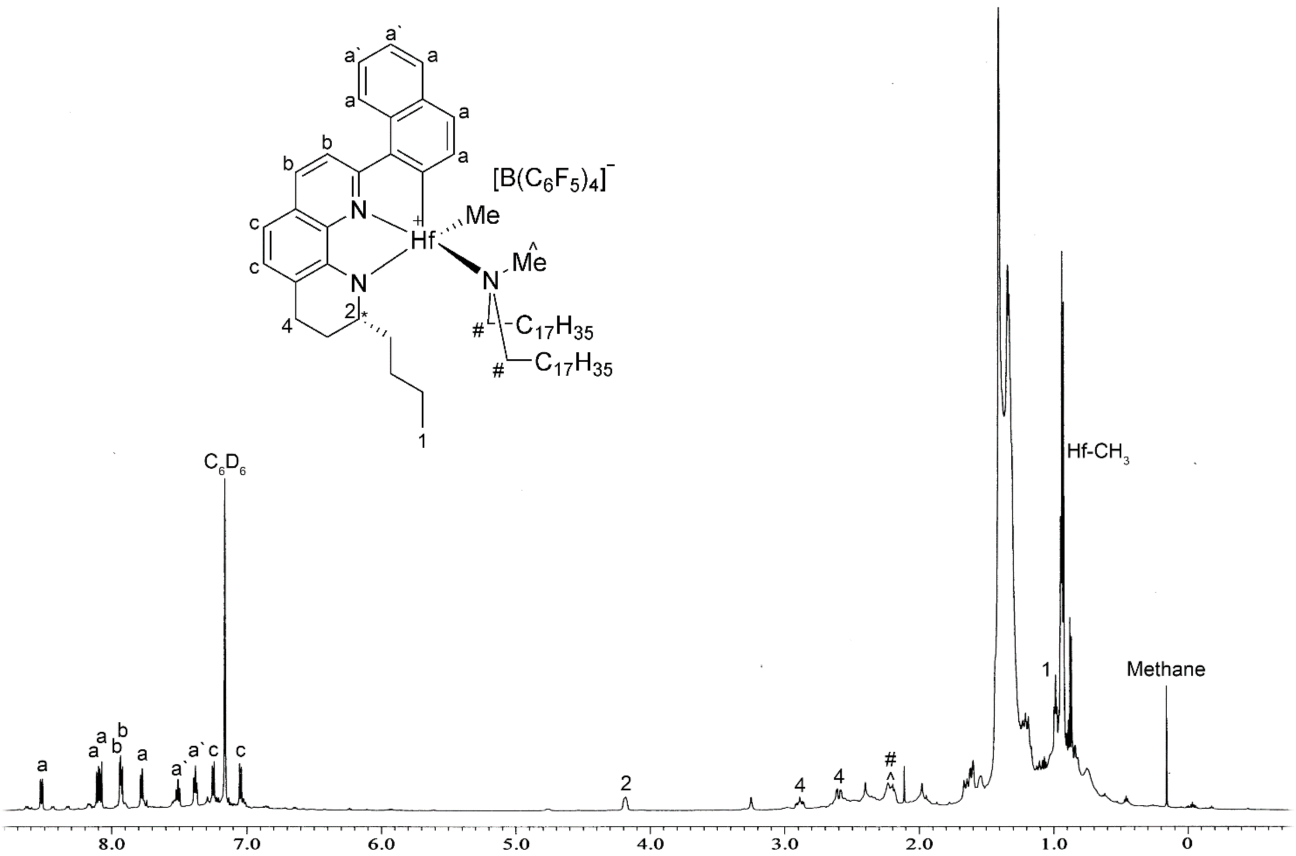

Figure S25. ^{1}H NMR spectrum recorded on the reaction of **16** with $[(C_{18}H_{37})_2N(H)Me]^{+}[B(C_6F_5)_4]^{-}$ at 1 h.

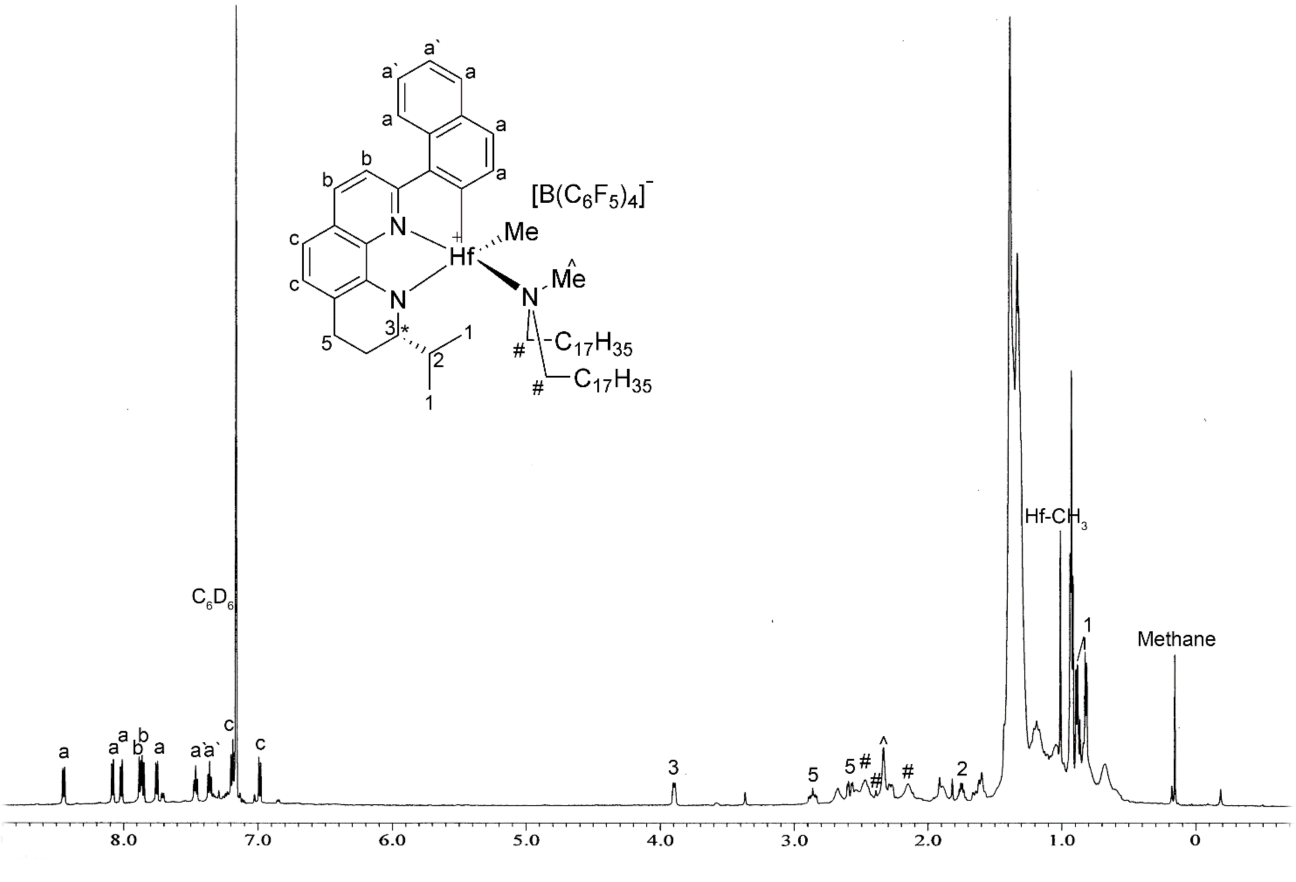

Figure S26. ^{1}H NMR spectrum recorded on the reaction of **16** with $[(C_{18}H_{37})_2N(H)Me]^+[B(C_6F_5)_4]^-$ containing water at 1 h.

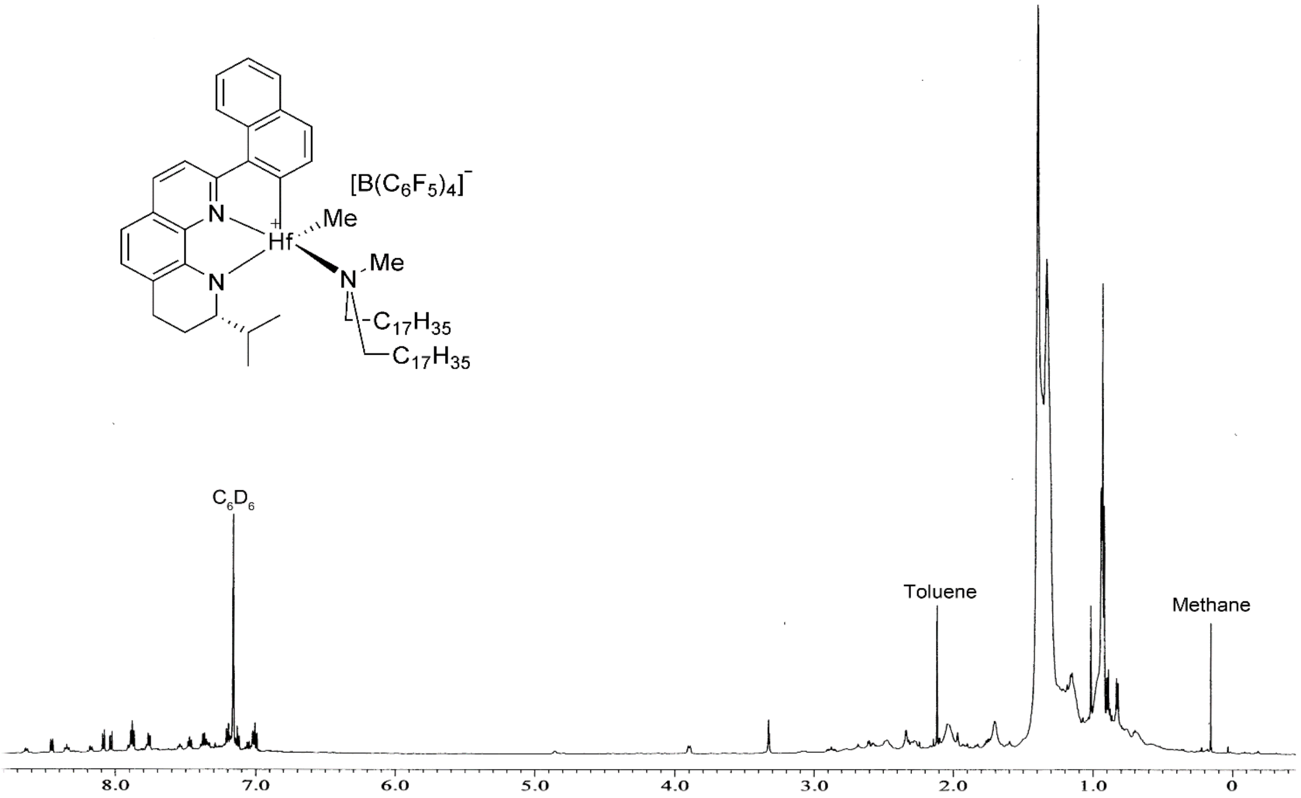

Figure S27. ^{1}H NMR spectrum of anhydrous $[(C_{18}H_{37})_2N(H)Me]^+[B(C_6F_5)_4]^-$ prepared in this work.

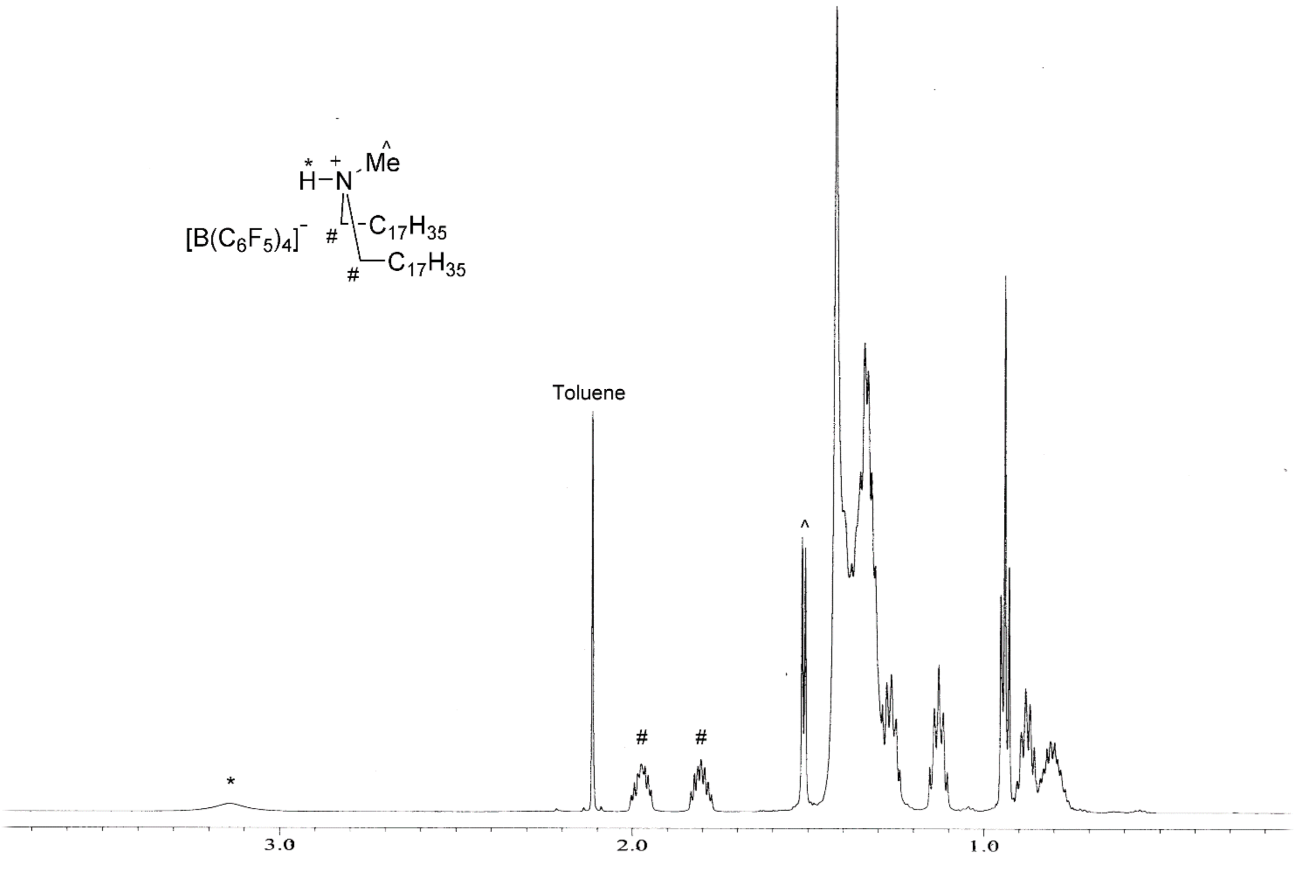

Figure S28. ^{1}H NMR spectrum of $[(C_{18}H_{37})_2N(H)Me]^+[B(C_6F_5)_4]^-$ containing water prepared by the method reported in patent.

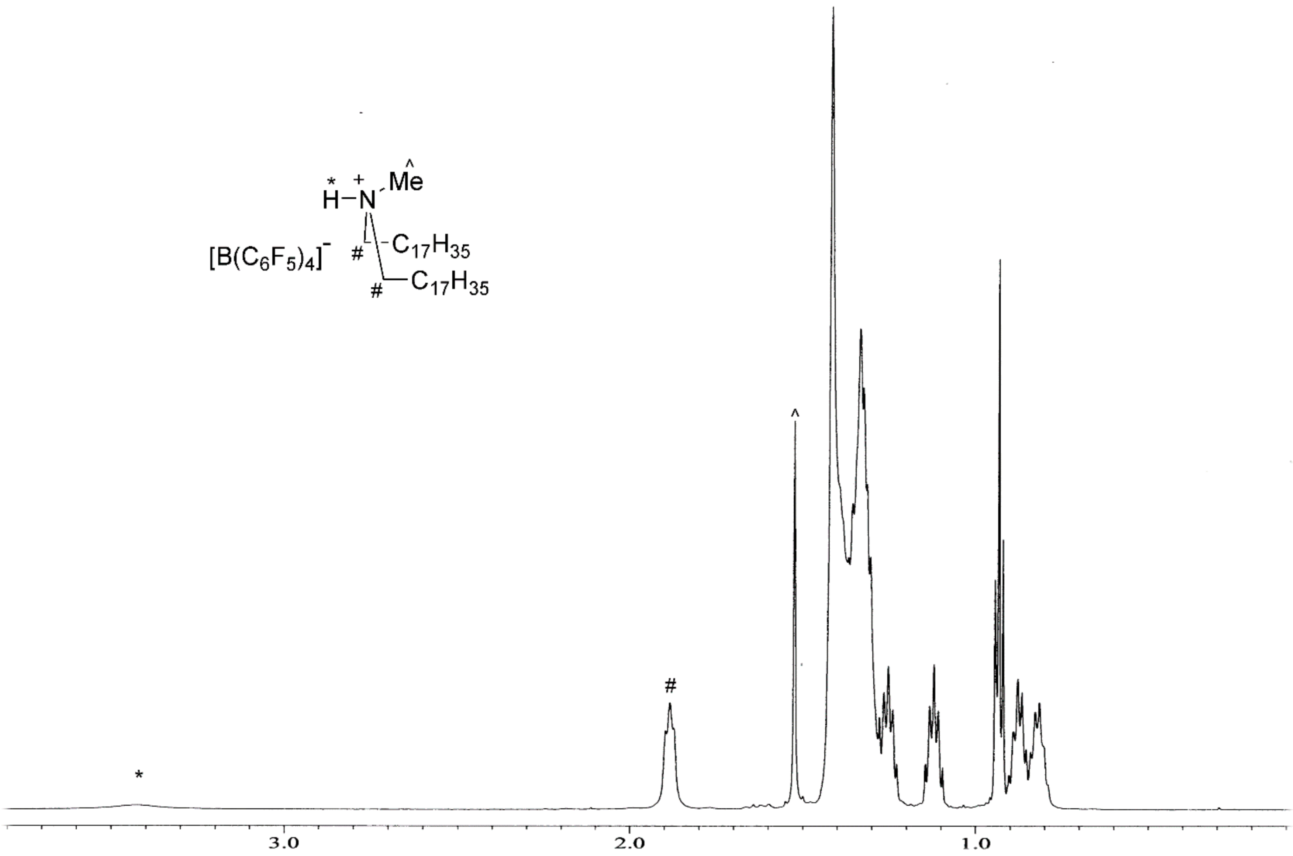

Figure S29. ^{1}H NMR spectrum of polymer (entry 3 in Table 1).

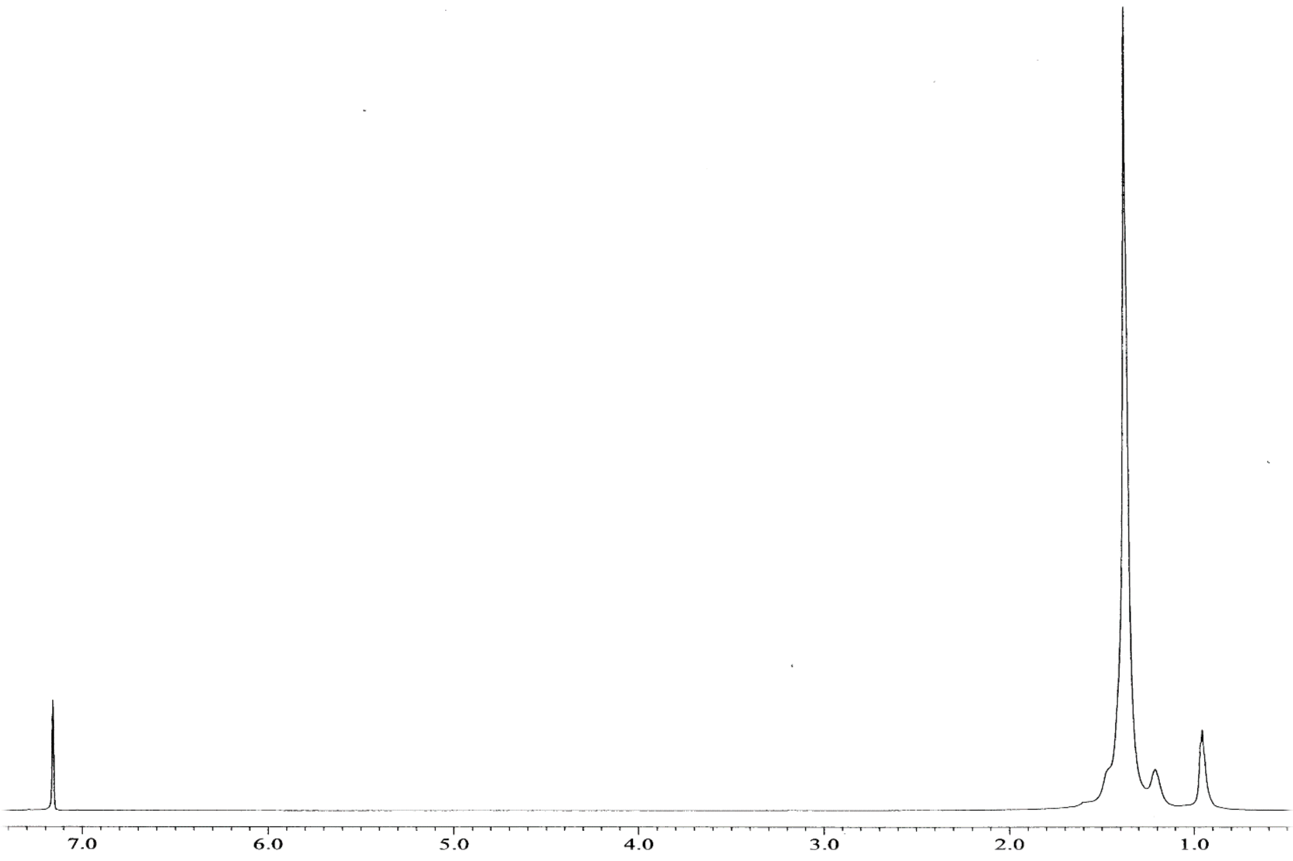

Figure S30. ^{1}H NMR spectrum of Polymer (entry 5 in Table 1).

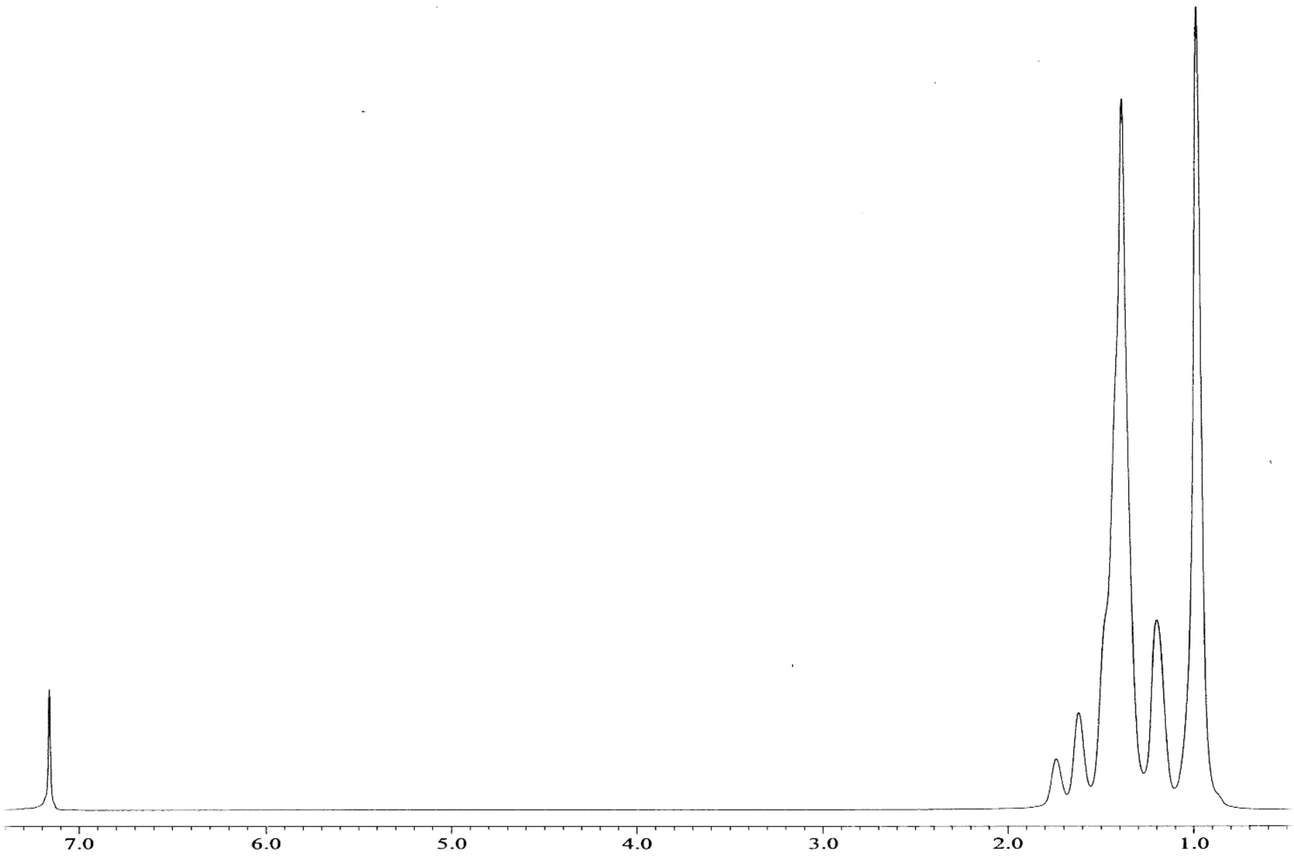

Figure S31. DSC Thermograms

<Entry 1 in Table 1>

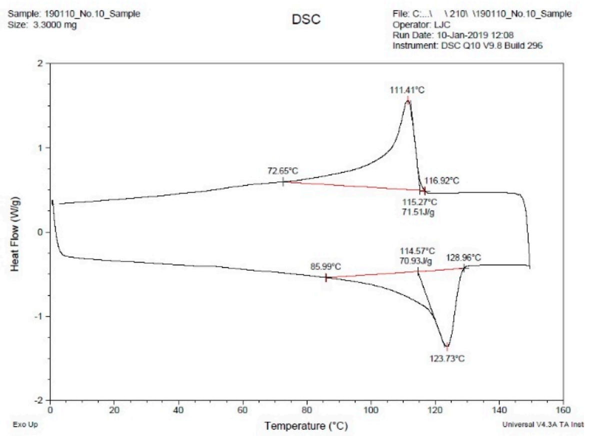

<Entry 2 in Table 1>

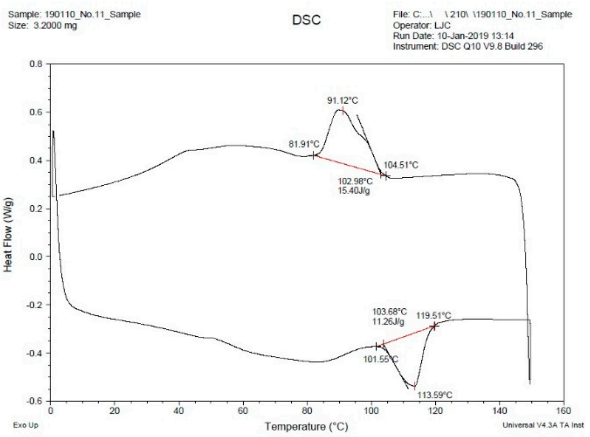

<Entry 3 in Table 1>

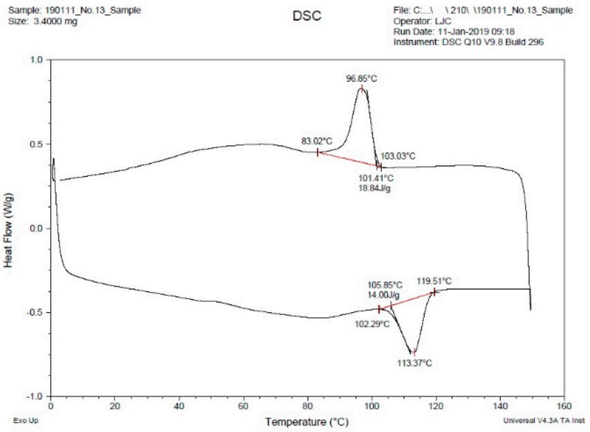

<Entry 4 in Table 1>

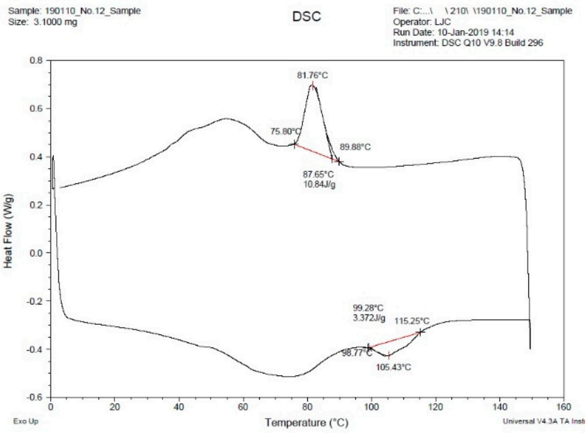

<Entry 5 in Table 1>

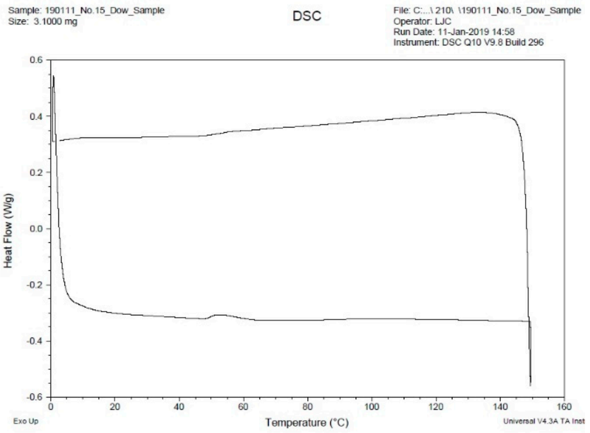

<Entry 6 in Table 1>

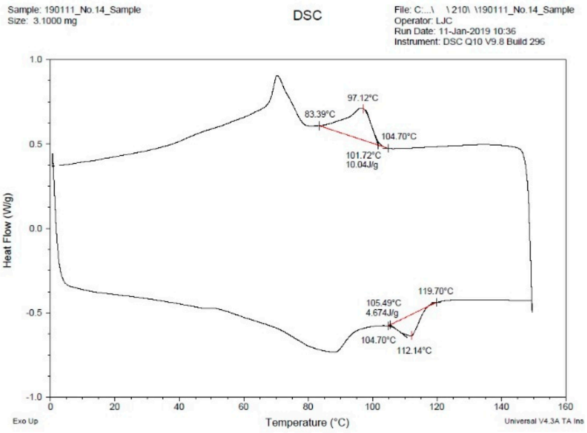

<Entry 7 in Table 1>

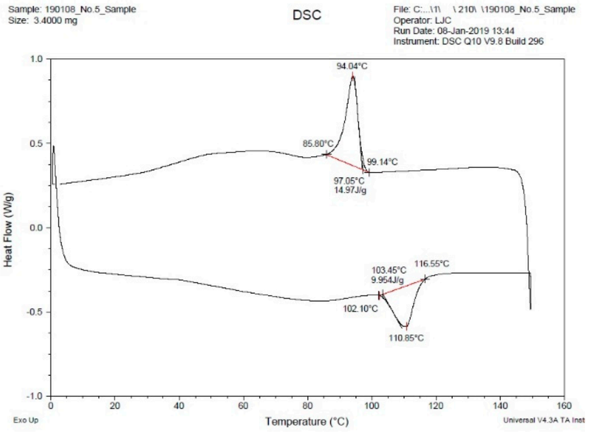

<Entry 8 in Table 1>

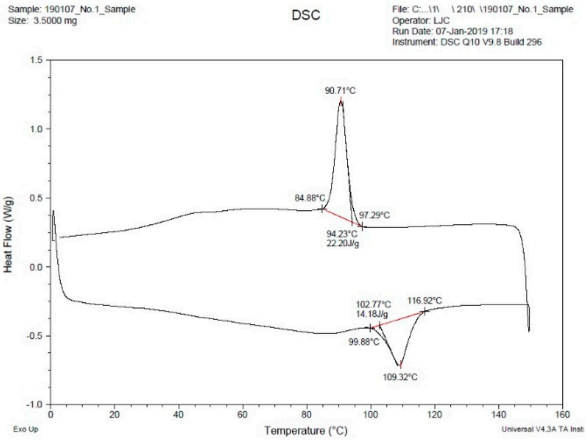

<Entry 9 in Table 1>

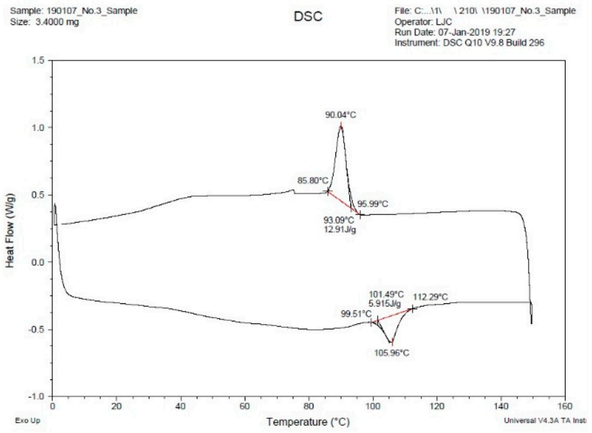

<Entry 10 in Table 1>

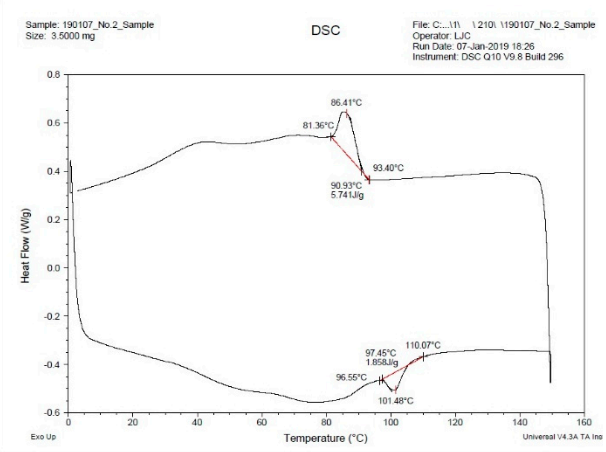

<Entry 11 in Table 1>

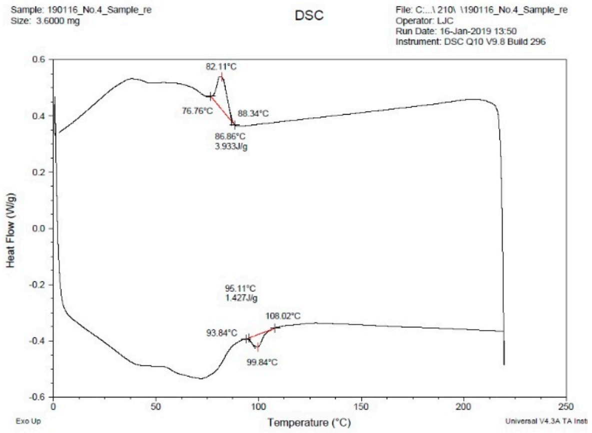

Supplement: Supplementary file 1 [file polymers-11-01093-s001.pdf]
